# Photoactivatable Ru(ii) polypyridyl complexes as dual action modulators of amyloid-beta peptide aggregation and Cu redox cycling

**DOI:** 10.1039/d5sc05593h

**Published:** 2025-10-01

**Authors:** Grace Leech, Alfredo Lopez Acosta, Samyadeb Mahato, Patrick C. Barrett, Rachel O. Hodges, Sherri A. McFarland, Tim Storr

**Affiliations:** a Department of Chemistry, Simon Fraser University V5A-1S6 BC Canada tim_storr@sfu.ca; b Department of Chemistry and Biochemistry, The University of North Carolina at Greensboro Greensboro NC USA 27402; c Department of Chemistry and Biochemistry, The University of Texas at Arlington Arlington Texas USA 76019 sherri.mcfarland@uta.edu

## Abstract

The misfolding and aggregation of the amyloid-β (Aβ) peptide is a major hallmark of Alzheimer's disease (AD), yet therapeutic strategies targeting this process have faced long-standing challenges related to efficacy and specificity. Here, we investigate two photoactivatable Ru(ii) polypyridyl complexes (RuP) that operate as dual-action modulators of AD pathology by addressing both Aβ aggregation and Cu-Aβ associated ROS generation. The RuP contain an extended planar imidazo[4,5-*f*] [1,10]phenanthroline ligand, which is important for pre-association with the Aβ peptide *via* hydrophobic and π–π interactions, as well as sterically hindered ligands 6,6′-dimethyl-2,2′-bipyridyl (6,6′-dmb) for RuP1 and 2,9-dimethyl-1,10-phenanthroline (2,9-dmp) for RuP2, which cause steric strain at the metal center. Photoactivation of the RuP results in loss of either a 6,6′-dmb or 2,9-dmp ligand exposing *cis*-exchangeable coordination sites for binding to the Aβ peptide, which immediately redirects the Aβ peptide away from its β-sheet-rich fibrillization pathway, promoting the formation of amorphous, off-pathway aggregates that exhibit increased sensitivity to proteolytic degradation. We find that the photoactivated RuP are closely associated with the amorphous aggregates, and that this is a common endpoint regardless of Aβ peptide aggregation state (monomer, oligomer, or fibril). Importantly, we show that the ejected ligands also inhibit the redox cycling and ROS generation of Cu-Aβ species. Together, these results highlight the potential of photoactivatable RuP as multifunctional therapeutic candidates, offering a rational approach to intercepting Aβ aggregation and Cu-mediated oxidative stress, and advancing the design of light-responsive treatments for neurodegenerative diseases.

## Abstract 2

Ligand photoejection from a strained Ru(ii) polypyridyl complex (RuP) results in dramatic modulation of amyloid-β (Aβ) peptide aggregation with the ejected ligand displaying additional benefits by limiting ROS generation *via* Cu sequestration.

## Graphical abstract

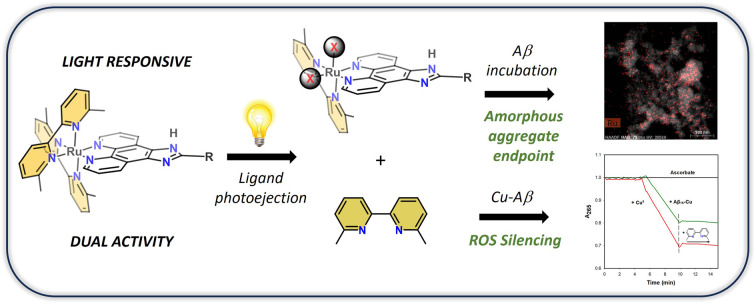

## Introduction

Dementia is a clinical syndrome marked by memory loss and significant cognitive decline, severely impairing an individual's ability to live independently.^1,2^ Alzheimer's disease (AD) accounts for 60–80% of these cases, making it the most prevalent progressive neurodegenerative disorder and the fifth leading cause of death among individuals aged 65 or older. The global burden of AD is anticipated to rise sharply due to increasing life expectancy.^3^

While the cause of AD continues to be investigated, its hallmark pathologies (*i.e.*, oxidative stress, tau protein aggregation, and amyloid-beta (Aβ) accumulation) have long been established.^4,5^ The amyloid hypothesis, proposed more than three decades ago, suggests that Aβ aggregation triggers a neurotoxic cascade.^6,7^ Aβ peptides arise from the proteolytic processing of the amyloid precursor protein (APP) and range from 38–43 residues in length. Aβ_1–40_ is the most abundant isoform (∼90%), while Aβ_1–42_ (∼9%) is highly prone to aggregation and considered the most neurotoxic.^8–12^ The self-assembly of Aβ into oligomers and fibrils disrupts synaptic connections, leading to neuronal loss and ultimately cell death.^13–18^ Although a number of drugs have been approved for AD treatment,^19–21^ these therapies do not halt or reverse disease progression. At best, they offer temporary symptomatic relief or modestly slow cognitive decline, highlighting the urgent need for effective disease-modifying treatments. The recent FDA-approval of monoclonal antibodies targeting Aβ peptide aggregation^22,23^ has highlighted the importance of the amyloid hypothesis and renewed interest in the development of small molecule agents that target this pathway.^24^

Oxidative stress is prevalent in AD, with early pathological changes indicative of oxidative damage.^25^ This oxidative stress is linked to a number of factors, including impaired energy metabolism, and redox-cycling of metal ions (Fe, Cu) in metal-containing Aβ peptide aggregates.^26–29^ Aβ plaques have been shown to contain increased concentrations of Cu^II^ (0.4 mM), Fe^III^ (0.9 mM), and Zn^II^ (1 mM), in comparison to normal tissue,^12,30,31^ and metal ion binding can modify the aggregation pattern, and initiate the production of reactive oxygen species (ROS).^32–34^ For example, studies have shown that Cu^II^ has a high affinity for the Aβ peptide (*K*_d_ ∼ 10^−10^ M),^35,36^ and Cu-Aβ species promote toxic catalytic ROS production by reducing O_2_ and generating the superoxide anion (O_2_˙^−^), hydrogen peroxide (H_2_O_2_), and hydroxyl radical (˙OH) *via* Fenton-like chemistry.^37–41^ As such, targeting this interaction by either disrupting metal–Aβ binding or by sequestering dysregulated metal ions is an attractive therapeutic approach.^42,43^

Given the complexity of AD, there is growing interest in therapeutic agents that address multiple pathological features simultaneously. In one such approach, multifunctional metal complexes that both interfere with Aβ aggregation and target an additional hallmark of the disease have gained attention.^32,42–51^ Studies involving Pt^II^, Rh^III^ and Ir^III^ complexes have revealed that covalent binding combined with non-covalent π–π interactions, facilitated by ligands such as 2,2′-bipyridine and 1,10-phenanthroline, that can promote pre-association of the metal complex with Aβ, a mechanism that may overcome specificity limitations observed in other AD therapies.^52–58^

Light-responsive inorganic complexes have emerged as an intriguing approach for both monitoring and inhibiting Aβ aggregation, offering spatiotemporal control and minimal invasiveness.^57,59–66^ Ruthenium polypyridyl (RuP) complexes have received considerable attention, and have been successfully applied across DNA intercalation, solar energy conversion, photodynamic therapy, and protein binding due to their favourable photophysical, electronic, and biological properties.^67–71^ In the context of AD, RuP can be tailored to favour specific interactions with Aβ upon photoactivation, such as monitoring aggregation and the formation of specific aggregate morphologies,^72^ facilitating peptide oxidation *via*^1^O_2_ generation, and promoting covalent binding through ligand dissociation.^73,74^

**Fig. 1:**
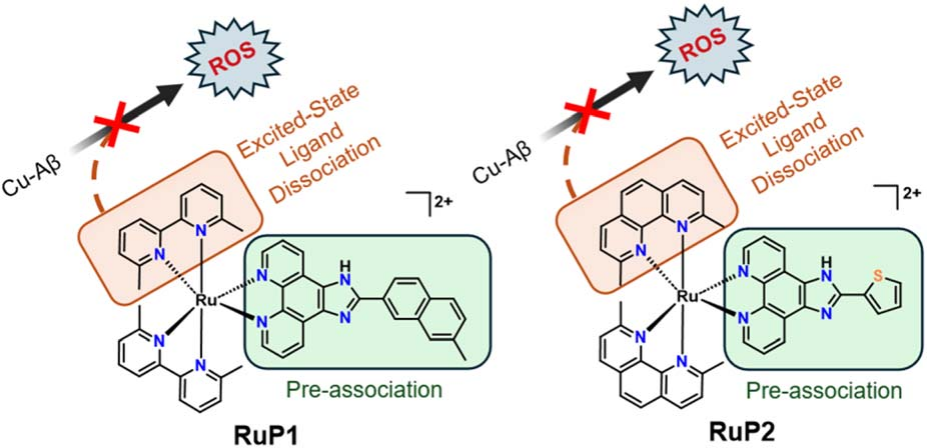
Photoactivatable RuP complexes used in this study. Photoactivation leads to selective ligand dissociation of 6,6′-dmb (RuP1) or 2,9-dmp (RuP2) to unmask exchangeable coordination sites for Aβ binding. Extended phenanthroline ligands are highlighted for their enhanced pre-association interactions. Released 6,6′-dmb or 2,9-dmp can interact with dysregulated metal ions (Fe, Cu, Zn) limiting Cu-Aβ associated ROS generation.

Herein, we investigate two photoactivatable RuP and their effects on Aβ aggregation and Cu redox cycling (Fig. 1). RuP1 and RuP2 were chosen for this study as they feature extended planar phenanthroline ligands that can facilitate pre-association interactions with Aβ,^72,74,75^ and 6,6′-dimethyl-2,2′-bipyridyl (6,6′-dmb) (RuP1) and 2,9-dimethyl-1,10-phenanthroline (2,9-dmp) for (RuP2) ligands that induce steric strain at the metal centre to promote ligand ejection when photoactivated. The photo-ejected ligands are hypothesized to provide further benefit by binding to Cu and Cu-Aβ species thereby limiting Cu-associated ROS generation. Such sterically encumbered bidentate ligands have shown utility in restricting Cu^II^/Cu^I^ redox processes when two such ligands are bound to Cu in a tetracoordinate manner.^76–78^ We show that photoactivation of RuP results in immediate changes to the Aβ aggregation process, forming more easily degraded amorphous aggregates, while the photo-ejected ligands provide additional benefit by limiting Cu-associated ROS generation.

## Results and discussion

### Photoactivation of RuP

In this study, RuP1 and RuP2 were chosen as they have previously been shown to dissociate one 6,6′-dmb or 2,9-dmp ligand respectively upon photoactivation (Fig. 1) due to the methyl groups that crowd the coordination sphere and distort the pseudo-octahedral geometry, thereby lowering the energy of the ^3^MC state and favouring relaxation *via* ligand dissociation.^68,71,73,79–81^ In addition, the planar aromatic phenanthroline ligands are known to exhibit a high affinity for amyloid aggregates,^60,72,82,83^ suggesting that RuP1 and RuP2 could be promising candidates for influencing the Aβ peptide aggregation pathway. In our previous work, a ruthenium complex bearing two 6,6′-dmb ligands and one bipyridine ligand, did not significantly modulate Aβ aggregation, which was attributed to its lack of extended hydrophobic ligands that can provide an enhanced interaction with Aβ.^74^ Finally, RuP1 and RuP2 exhibit limited toxicity under dark normoxic conditions (EC_50_ = 24.8 μM (RuP1) and EC_50_ = 185 μM (RuP2) in SK-MEL-28 cells).^84,85^ In addition, the stability of unactivated RuP1 and RuP2 under physiological conditions was confirmed by UV-vis spectroscopy, with no spectral changes observed after 24 h, indicating that the complexes remain intact in the absence of light (Fig. S1). We thus endeavoured to study how photoactivation of RuP1 and RuP2 influences the interaction of these complexes with the Aβ peptide and how this affects the peptide aggregation process.

Upon visible-light exposure, endpoints for ligand dissociation are reached at 43 J cm^−2^ for RuP1, and 101 J cm^−2^ for RuP2 (Fig. S3). The faster ejection of the 6,6′-dmb ligand from RuP1 is attributed to the greater flexibility of the bipyridine backbone as compared to 2,9-dmp ligand of RuP2, and the stronger σ-donation and π-accepting properties of the 2,9-dmp ligand. In both complexes, photoactivation selectively ejects the strain-inducing dimethyl-substituted bipyridine or phenanthroline ligand, affording readily exchangeable coordination sites for Aβ peptide binding (Fig. S4 and S5).

**Fig. 2:**
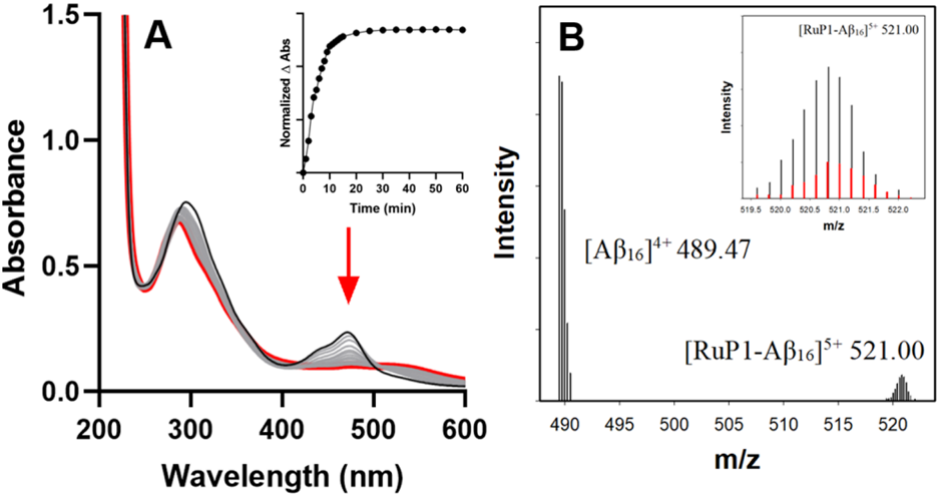
(A) UV-vis absorption spectra of RuP1 before (black) and after light activation conditions (grey) until an endpoint was reached (red). Conditions: RuP1 25 μM, PBS (0.01 M, pH 7.4), SOLLA 30 W LED at an irradiance of 48 mW cm^−2^. Inset: Absorbance change at 473 nm over time showing an endpoint for ligand release at 15 min for RuP1. (B) ESI-MS of Aβ_1–16_ (200 μM) in the presence of 1 equiv. of photoactivated RuP1 in NH_4_CO_3_ buffer (pH 9.0). Inset: Calculated isotopic pattern of RuP1-Aβ_1–16_ adduct in red.

To investigate binding, ESI-MS studies were performed with a 1 : 1 ratio of RuP and Aβ_1–16_, a hydrophilic peptide containing key metal-binding residues His^6/13/14^, which is less prone to aggregation allowing for clearer interpretation of binding interactions. In the absence of light, no adducts were observed, and only peaks for the intact RuP complexes and Aβ_1–16_ were detected (Fig. S6 and S7). However, upon photoactivation ESI-MS revealed Ru-Aβ_1–16_ adducts at *m*/*z* = 521.00 ([RuP1-Aβ_1–16_]^5+^) and *m*/*z* = 514.19 ([RuP2-Aβ_1–16_]^5+^) (Fig. 2, S8 and S9), with the calculated isotopic patterns confirming loss of the 6,6′-dmb and 2,9-dmp ligands and subsequent binding to Aβ_1–16_, showing that adduct formation occurs exclusively upon photoactivation.

### Influence of RuP on Aβ peptide aggregation

**Fig. 3:**
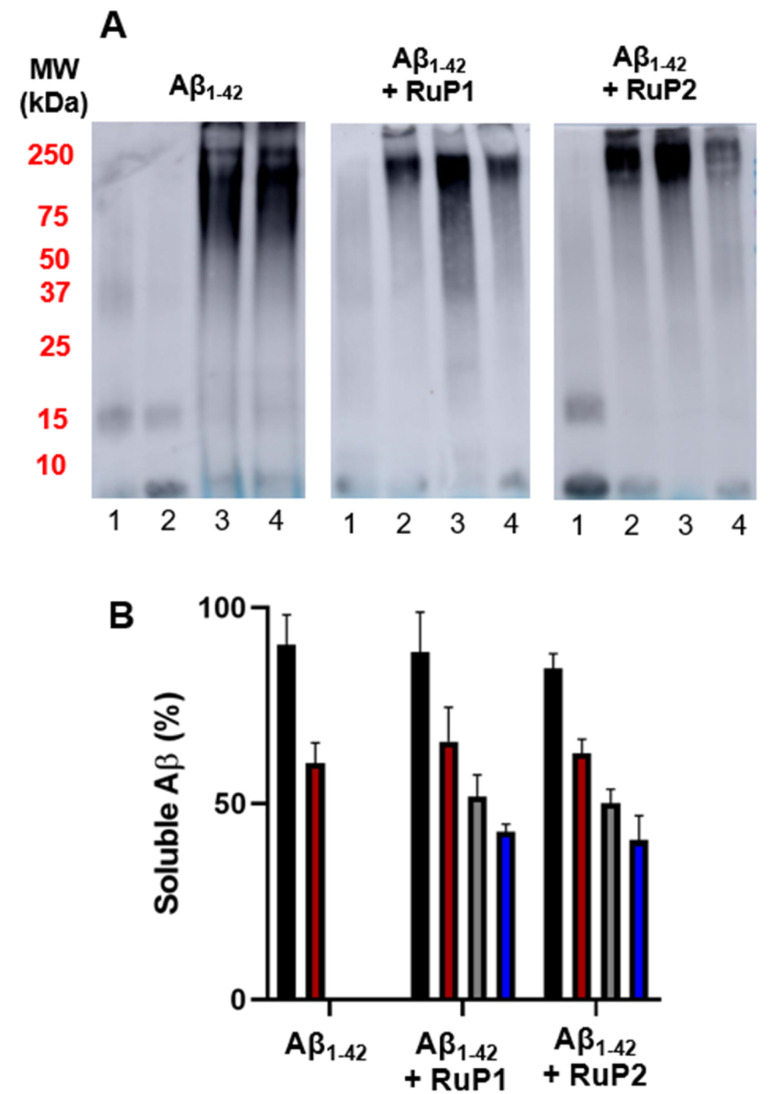
(A) Western blot of 25 μM Aβ_1–42_ in the absence and presence of 1 equiv. of RuP in PBS (0.01 M, pH 7.4) at incubation times 0 h and 24 h. Lane 1: 0 h unactivated, lane 2: 0 h photoactivated, lane 3: 24 h unactivated, lane 4: 24 h photoactivated. (B) BCA assay of 25 μM Aβ_1–42_ and 1 equiv. of RuP in PBS (0.01 M, pH 7.4) at incubation times 0 h with (grey) and without (black) photoactivation and 24 h with (blue) and without (red) photoactivation. Absorbance was measured at 562 nm.

Due to the evidence of photo-induced adduct formation revealed by ESI-MS, we next investigated the influence of RuP on the Aβ peptide aggregation pathway. The Aβ_1–42_ peptide was chosen for these studies due to its high propensity to aggregate and toxicity.^8–12^ Although similar Ru polypyridyl complexes with extended planar hydrophobic ligands have been shown to exhibit noncovalent interactions with the Aβ peptide^60,72,82,83^ and, in some cases, influence its aggregation pathway,^86,87^RuP (25 μM) did not significantly alter the aggregation pattern of the Aβ_1–42_ peptide in the dark relative to the peptide alone after a 24 h incubation at 37 °C (Fig. S10) according to a light scattering turbidity assay. We next examined the influence of RuP on Aβ_1–42_ aggregation using gel electrophoresis and western blotting. At 0 h, Aβ_1–42_ (25 μM) was primarily present as monomers and dimers (low MW species), with higher MW species appearing after 24 h, consistent with prior reports (Fig. 3A).^88–90^ The addition of 1 equiv. unactivated RuP caused minimal changes, in agreement with turbidity results (Fig. 3 and S10). In contrast, photoactivation of RuP1 and RuP2 caused an immediate shift in the Aβ_1–42_ aggregation pathway from monomers/dimers to high MW aggregates (Lane 2, Fig. 3A), bypassing the intermediate oligomers (10–100 kDa) that have been associated with significant toxicity.^16,17^ Notably, these high MW species remained largely unchanged over the 24 h time course (Lane 4), indicating that photoactivation of RuP results in an immediate and lasting effect on Aβ_1–42_ aggregation. Interestingly, this effect is not observed when RuP is photoactivated before incubation with Aβ_1–42_ (Fig. S11), suggesting that RuP-Aβ pre-association is an important factor in the observed aggregation results. Finally, we investigated if the free ligands alone (that would be present upon photoejection) could influence Aβ aggregation, however, western blot analysis at 0 h and 24 h confirmed no observable effect on the peptide aggregation process (Fig. S12).

To further investigate the impact of RuP photoactivation on Aβ_1–42_ solubility, a bicinchoninic acid (BCA) assay was performed. Prior to measurement, the samples were centrifuged (14 000*g*, 5 min) to remove insoluble aggregates.^91^ The results reveal a *ca.* 40% reduction in soluble Aβ_1–42_ after a 24 h incubation time (Fig. 3B), for both peptide alone and for peptide in the presence of 1 equiv. unactivated RuP. Notably, immediately after photoactivation there is a >55% decrease in peptide solubility for both RuP1 and RuP2, which agrees with the formation of large MW aggregates observed in the gel experiment (Fig. 3A). No further decrease in the solubility of the peptide takes place after 24 h, suggesting that photoactivation coincides with peptide aggregation/precipitation, with minimal changes occurring after further incubation. Overall, the gel electrophoresis/western blotting experiments align with the BCA assay results, confirming that photoactivation of RuP rapidly redirects Aβ_1–42_ aggregation toward insoluble, high molecular weight species.

**Fig. 4:**
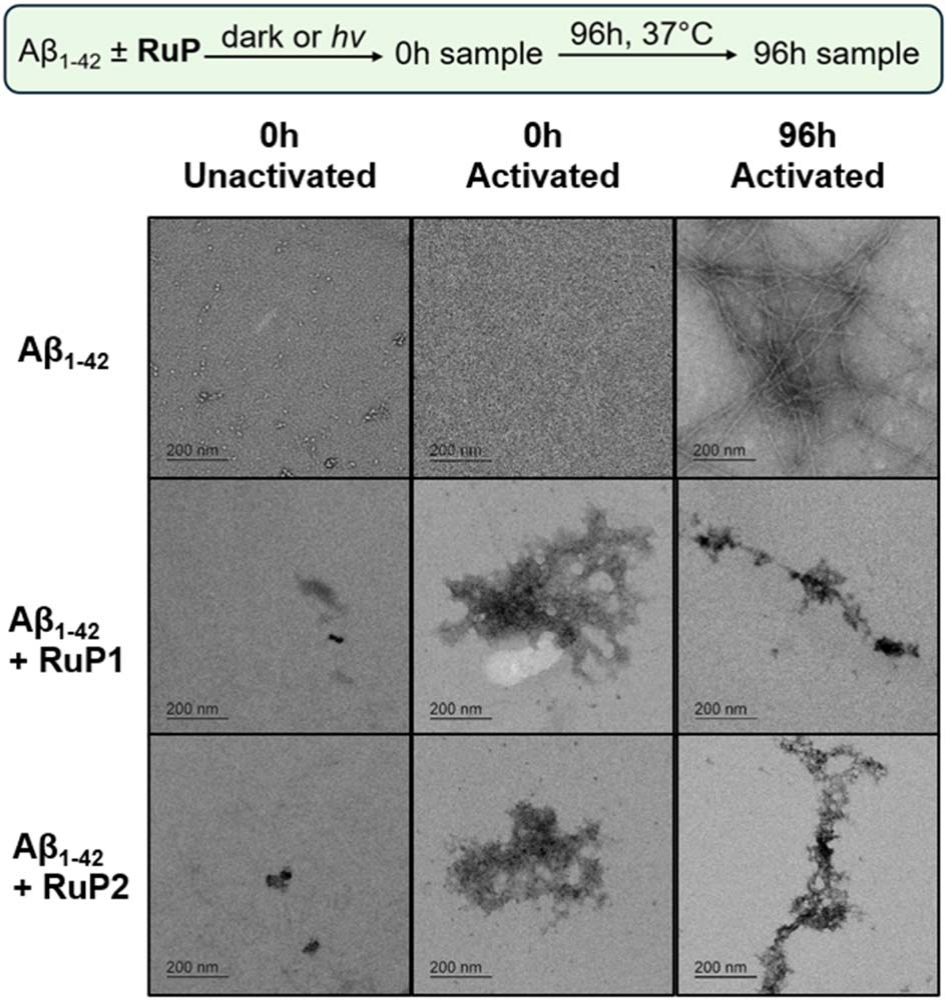
TEM images of 25 μM Aβ_1–42_ alone and in the presence 1 equiv. of RuP1 and RuP2 at 0 h unactivated, 0 h activated and 96 h activated. Scale bar = 200 nm.

While gel electrophoresis and western blotting provides information on soluble high MW Aβ_1–42_ aggregates, transmission electron microscopy (TEM) can be used to characterize larger insoluble Aβ_1–42_ aggregates, that are not able to efficiently penetrate the gel matrix. The combination of these methods provides a more comprehensive understanding of the Aβ_1–42_ aggregation pathway under our conditions. As expected for the Aβ_1–42_ sample at 0 h, regardless of photoactivation, no aggregates were observed (Fig. 4). Similarly, in the presence of 1 equiv. unactivated RuP there was no change observed at 0 h. Interestingly, in the presence of 1 equiv. photoactivated RuP at the 0 h timepoint, diffuse amorphous aggregates are observed. Incubation of Aβ_1–42_ alone for 96 h led to the formation of large β-sheet rich fibrillar species, agreeing with previous reports (Fig. 4).^74,92^ After 96 hours, Aβ_1–42_ incubated with 1 equiv. of photoactivated RuP remains as amorphous aggregates, indicating that their immediate formation represents a stable endpoint in the aggregation pathway as they do not rearrange into ordered fibrillar structures (Fig. 4). The TEM data is consistent with the gel electrophoresis experiments, revealing that photoactivation of RuP immediately promotes the formation of diffuse amorphous aggregates, modulating the known aggregation pathway which produces mature β-sheet fibrils.

We also investigated if photoactivated RuP could change the aggregation profile of intermediate MW species, including toxic oligomers.^16,17^ Aβ_1–42_ aggregation after 16 h at 4 °C with constant agitation revealed a range of species such as monomers, dimers, trimers, and higher molecular weight aggregates (Fig. S13). Upon RuP photoactivation, high molecular weight species exclusively formed, identified by TEM as amorphous aggregates (Fig. S14). This closely parallels results observed with monomeric Aβ_1–42_ (above) indicating that ligand ejection and binding direct aggregation toward amorphous, off-pathway species, bypassing the toxic oligomeric stage regardless of when they are introduced.

**Fig. 5:**
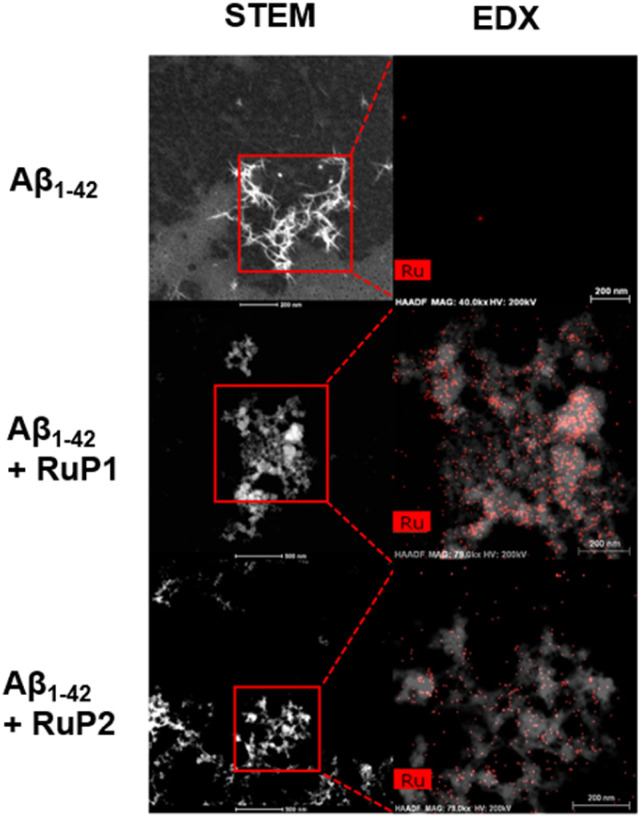
High-angle annular dark field (HAADF) STEM & EDX of 25 μM Aβ_1–42_ alone and in the presence of 1 equiv. of photoactivated RuP and then incubated for 96 h. Red box represents the area used for elemental mapping of Ru. Scale bar = 200 nm.

To further characterize the amorphous aggregates formed after photoactivation of RuP in the presence of Aβ_1–42_, we used high-resolution TEM coupled with Energy-Dispersive X-ray Spectroscopy (EDX) to analyze their structure and elemental composition. This approach allowed us to map Ru within the aggregates, providing further insight into the role of RuP in modulating Aβ_1–42_ aggregation and aggregate morphology. As expected, Aβ_1–42_ alone showed no Ru content in fibrils after 96 h (Fig. 5). In the presence of 1 equiv. unactivated RuP, Ru was dispersed over the sample grid and not closely associated with the peptide aggregate (Fig. S15). In contrast, photoactivated RuP show significant overlap of the Ru signal and the amorphous aggregates, with RuP1 showing the most pronounced effect. These results indicate a close association of the photoactivated complexes with the amorphous aggregates, providing further insight into how RuP binding to Aβ_1–42_ influences both the Aβ_1–42_ peptide aggregation pathway and resulting aggregate morphology.

### Interaction of RuP with Aβ peptide fibrils

**Fig. 6:**
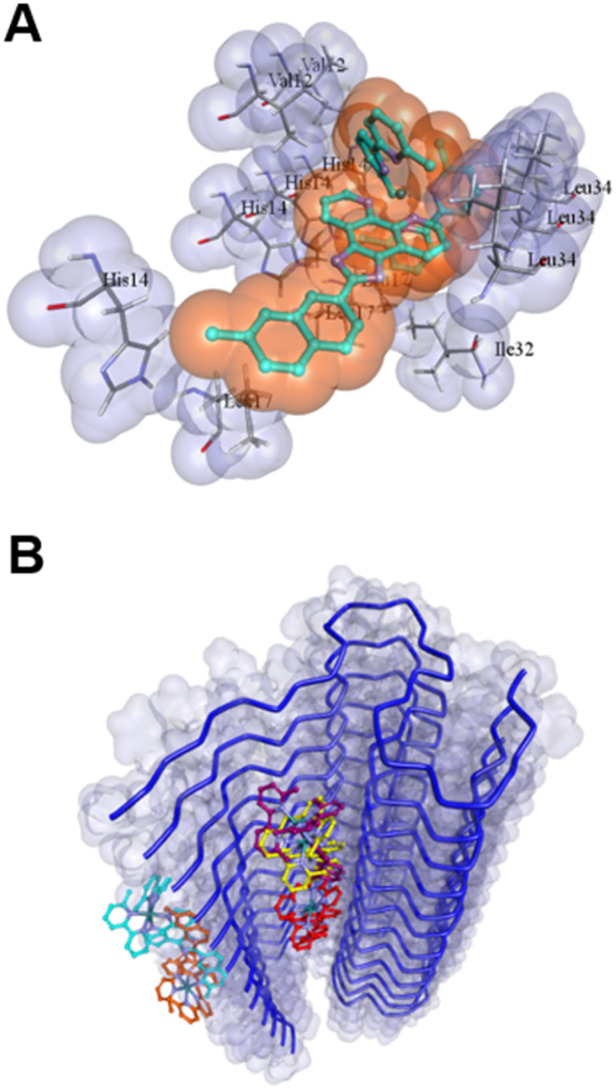
(A) Molecular docking of a representative binding mode of unactivated RuP1 to PDB structure 2MXU identifying potential interactions with specific amino acids Val^12^, His^14^, Ile^32^, Leu^34^. (B) The nine most stable binding modes of unactivated RuP1 to PDB structure 2MXU.

To investigate potential RuP interactions with Aβ_1–42_ fibrils, molecular docking was performed using PDB structures 5OQV^93^ and 2MXU,^94^ representing single- and double-symmetry fibril surfaces, respectively. The 2MXU structure, with its hydrophobic cleft and 12 β-strand filaments, offers extensive surface area but lacks the Aβ_1–10_ region, which is present in the shorter 5-strand 5OQV fibril. Using both structures allowed a more comprehensive analysis of RuP binding modes (Fig. 6, S16–S18). Docking studies revealed multiple potential binding sites, with RuP1 and RuP2 displaying comparable binding scores across both PDB structures (Tables S1–S4). The diversity of binding sites are likely of moderate affinity, as EDX analysis showed minimal interaction between unactivated RuP and the Aβ_1–42_ peptide, as indicated by the uniform distribution of Ru across the sample grid (Fig. S15). A representative binding mode for both RuP1 and RuP2 on 2MXU involves interactions with His^14^ residues, suggesting close association with a key metal-ion binding site (Fig. 6A and S18), with significant interactions between the hydrophobic extended phenanthroline ligand and the Aβ_1–42_ fibrils. The presence of several accessible binding sites may facilitate enhanced covalent binding upon photoactivation.

**Fig. 7:**
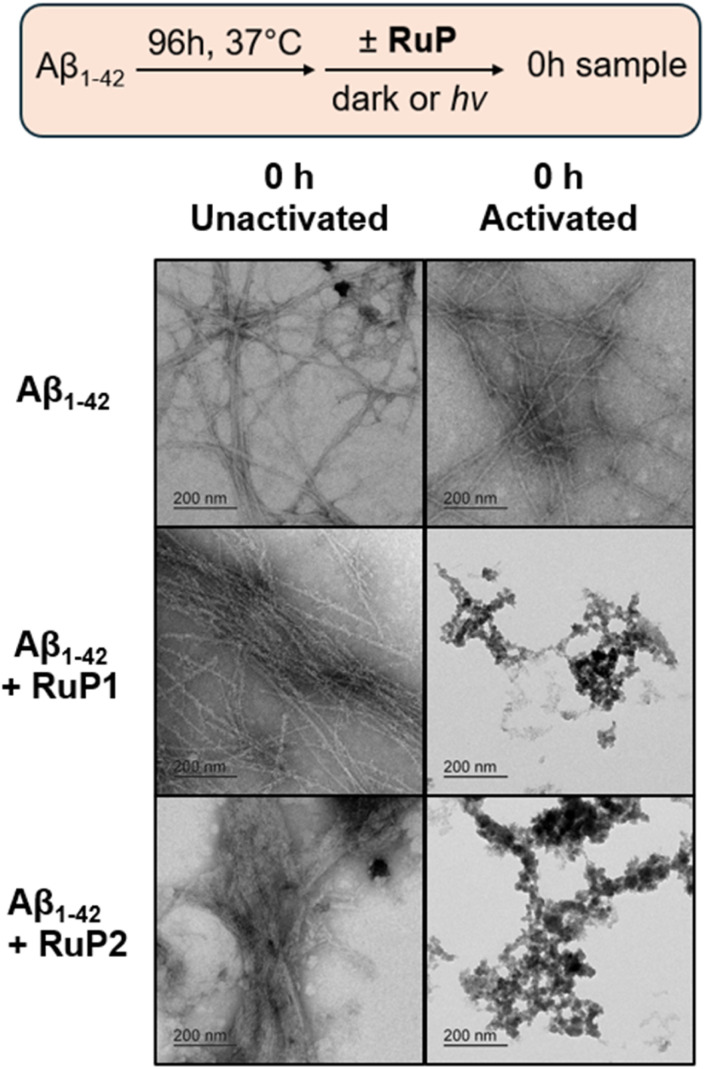
Influence of 1 equiv. of RuP1 and RuP2 on the morphology of pre-formed Aβ_1–42_ fibrils (25 μM) before and immediately after photoactivation using TEM. Scale bar = 200 nm.

To investigate if the RuP can change the morphology of Aβ_1–42_ fibrils, we incubated pre-formed fibrils with unactivated and activated RuP and investigated for structural changes by TEM. In the presence of 1 equiv. of unactivated RuP structured aggregates were maintained. Remarkably, in the presence of 1 equiv. of either photoactivated RuP1 or RuP2, an immediate morphology change from structured fibrillar aggregates to amorphous aggregates are observed (Fig. 7). This once again reinforces that photoactivation is required to observe a change in morphology of the Aβ peptide, and in addition highlights that amorphous aggregates are a common endpoint regardless of where photoactivated RuP is introduced along the Aβ peptide aggregation pathway.

**Fig. 8:**
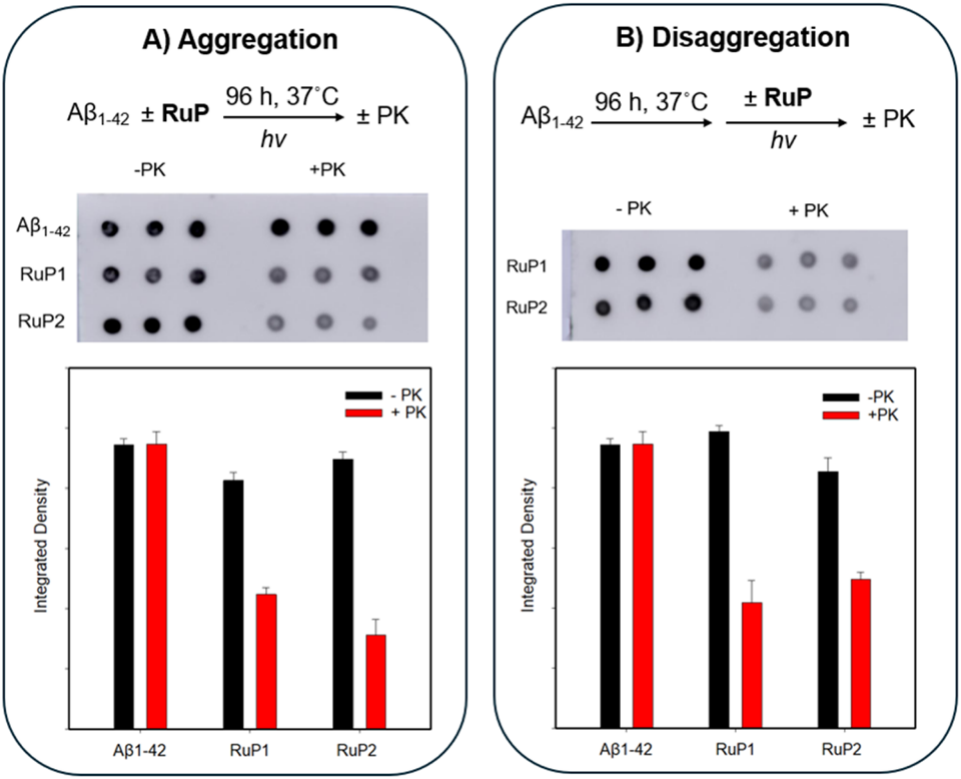
(A) Proteinase-K immunodot blot assay of Aβ_1–42_ alone (25 μM) after 96 h and in the presence of 1 equiv. photoactivated RuP, with and without proteinase-K (5 μM) (top) using 6E10 antibody. Signal response plotted by integrated density using Image J software (bottom). (B) Proteinase K immunodot blot assay of Aβ_1–42_ (25 μM) pre-aggregated for 96 h, followed by addition of 1 equiv. photoactivated RuP with and without proteinase (5 μM). Signal detected and quantified as in (A).

The Aβ peptide is cleared from the brain through enzymatic degradation, transport across the blood–brain barrier, and bulk flow of interstitial and cerebrospinal fluid.^95^ Importantly, the structure of the Aβ aggregate influences both their pathogenicity and their susceptibility to clearance mechanisms, with certain species, like ordered fibrils, being resistant to proteolysis,^96^ while amorphous aggregates have been reported to be more easily degraded and generally associated with lower pathogenicity compared to β-sheet-rich fibrils.^91,94,97,98^ This suggests that altering the aggregation pathway to favour amorphous species, as achieved with photoactivated RuP, could be a promising therapeutic approach. However, our EDX results show significant incorporation of the RuP complexes into the Aβ aggregates, which may inhibit enzymatic degradation. To assess the susceptibility to enzymatic degradation of the amorphous aggregates formed with photoactivated RuP, we performed a proteinase-K (PK) assay,^99,100^ after incubating Aβ_1–42_ (25 μM) alone, or with 1 equiv. photoactivated RuP1 or RuP2 for 96 h followed by addition of PK (5 μM). In the absence of PK, no notable differences were observed between Aβ_1–42_ and RuP treated samples, as expected. However, when PK was added, immunodot blot analysis revealed a marked loss of signal for the amorphous aggregates generated by photoactivated RuP1 and RuP2, indicating increased protease sensitivity (Fig. 8A), despite the presence of significant photoactivated RuP in the sample as indicated by EDX (Fig. 5). Integrated density quantification confirmed that both RuP1 and RuP2 induced amorphous aggregates were similarly susceptible to PK degradation, supporting their enhanced clearance potential. The assay was also applied to pre-formed Aβ_1–42_ fibrils (96 h), where samples of fibrils alone were treated with PK, and fibrils incubated with 1 equiv. RuP underwent photoactivation followed by PK treatment (Fig. 8B). Consistent with earlier findings, increased protease sensitivity was observed, indicating that the amorphous aggregates formed upon RuP photoactivation, regardless of the initial aggregation state, are more susceptible to proteinase K degradation. Notably, enzymatic degradation was not inhibited by the presence of RuP, which is advantageous for therapeutic strategies aimed at facilitating the clearance of pathogenic aggregates.

### Ligand photoejection inhibits Cu-Aβ associated ROS generation

Cu-Aβ species are known to produce ROS, including the superoxide anion, hydrogen peroxide, and the hydroxyl radical, and such ROS generation is proposed to be a major contributor to the toxicity of Aβ aggregates.^39,40,45,101,102^ Previous research has shown that the bipyridine ligand scaffold can inhibit Aβ peptide aggregation in the presence of Cu and Zn ions,^103^ and limit Cu-Aβ toxicity in cell lines.^104,105^ Additional research on the phenanthroline ligand scaffold has shown its utility in inhibiting the toxicity of Cu/Zn-Aβ species and reduce the amyloid burden in a mouse model.^106,107^ Herein, we aimed to understand if the photoejected ligands (6,6′-dmb and 2,9-dmp, Fig. 1) could limit the redox cycling and ROS generation of Cu-Aβ species, providing an additional mechanism of action for the RuP.

ESI-MS was initially used to assess whether the photo-ejected ligands could compete with the Aβ peptide for Cu. As expected, incubation of unactivated RuP with pre-formed Cu-Aβ_1–16_ did not show any ligand release or Cu complexation by 6,6′-dmb or 2,9-dmp (Fig. S19 and S20). However, upon photoactivation, Cu adducts [Cu(6,6′-dmb)_2_]^+^ (*m*/*z* = 431.14) for RuP1 and [Cu(2,9-dmp)_2_]^+^ (*m*/*z* = 479.13) for RuP2 were identified (Fig. S21 and S22).

**Fig. 9:**
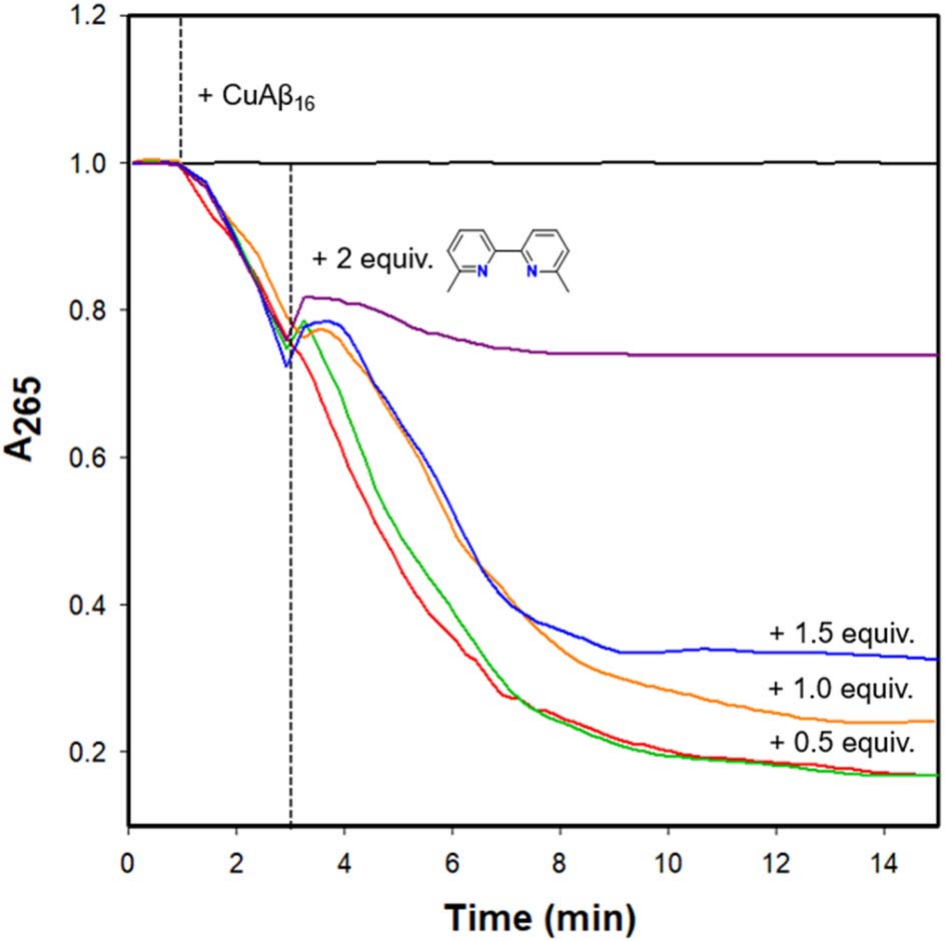
Ascorbate consumption assay measuring absorbance at 265 nm of ascorbate alone (black), and upon addition of Cu-Aβ_1–16_ (red) at 1 min (---), followed by addition of 0.5 (green), 1.0 (orange), 1.5 (blue) and 2.0 (purple) equivalents of 6,6′-dmb at 3 min (---). Conditions: [Asc] = 200 μM, [CuCl_2_] = 25 μM, [6,6′-dmb] = 12.5–50 μM, [Aβ_1–16_] = 25 μM, PBS (0.01 M, pH 7.4).

To evaluate the impact of photoactivated RuP on Cu-Aβ redox cycling and ROS generation, we first monitored ascorbate (Asc) oxidation by measuring the decay of its absorbance band at 265 nm.^108^ Asc remained stable over 15 min, but upon addition of Cu^II^-Aβ_1–16_ at the 1 min timepoint, Asc consumption is evident (Fig. 9). Interestingly, upon subsequent addition of 0.5–1.5 equiv. of 6,6′-dmb (Fig. S23 for 2,9-dmp) at the 3 min timepoint results in little to no effect on Asc consumption. However, the addition of two equiv. of 6,6′-dmb to the Cu^II^-Aβ_1–16_ reaction solution immediately halted the ascorbate oxidation process, indicating that two equiv. of the ligand is required to suppress Cu^II^/Cu^I^ redox activity *via* chelation and/or ternary complex formation. In addition, 2 equiv. of ligand is needed to inhibit Asc oxidation in the case of Cu alone (Fig. S24), which is consistent with restricted Cu^II^/Cu^I^ redox cycling when two sterically-hindered bidentate ligands (such as 6,6′-dmb) are coordinated to the Cu centre.^76–78^

**Fig. 10:**
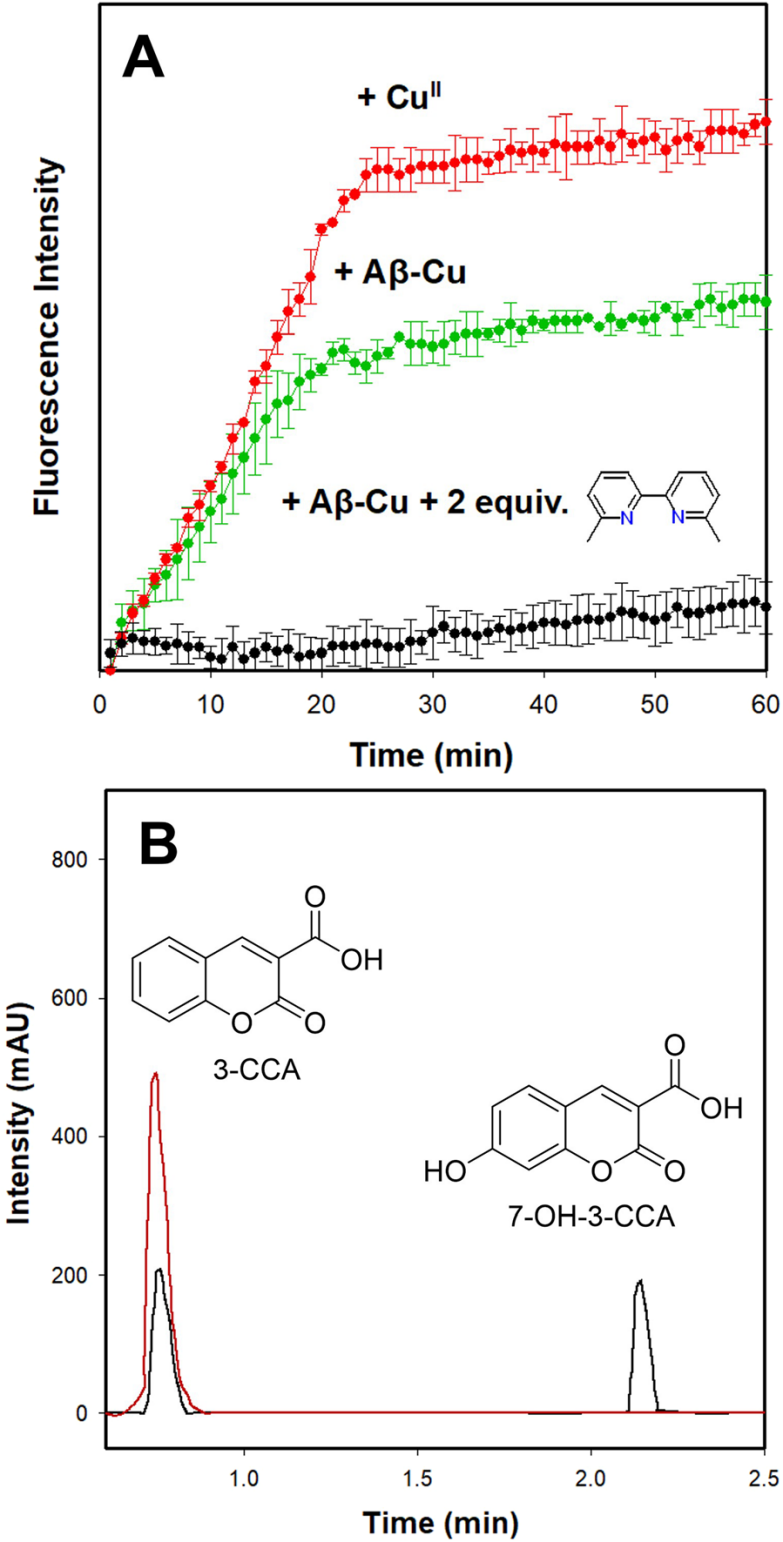
(A) CCA assay for ˙OH detection measured by fluorescence, *λ*_ex_ = 390 nm and *λ*_em_ = 450 nm. (B) HPLC of CCA assay in the presence of RuP1 unactivated (black) and activated (red) in PBS (0.01 M, pH 7.4). Conditions: [Asc] = 200 μM, [CCA] = 200 μM, [CuCl_2_] = 25 μM, [RuP1 or 6,6′-dmb] = 50 μM, [Aβ_1–16_] = 25 μM.

To confirm that this effect translates to inhibiting ROS generation, a 3-coumarin carboxylic acid (CCA) assay was performed (Fig. 10A). The CCA assay detects the formation of the ˙OH radical from the reaction of Cu^I^ with H_2_O_2_*via* the reaction of 3-coumarin carboxylic acid (3-CCA) with ˙OH to form the fluorescent 7-hydroxy-3-coumarin-carboxylic acid (7-OH-3-CCA).^109^ Consistent with previous reports,^29^ ˙OH production in the presence of Cu^II^ is rapid, with a lower but still significant response for Cu^II^-Aβ (Fig. 10A). Addition of 2 equiv. of 6,6′-dmb or 2,9-dmp to Cu^II^-Aβ completely abolishes ˙OH production, suggesting that the photoejected ligands suppress this form of ROS generation (Fig. 10A and S25A). Addition of RuP to the CCA assay resulted in interference, therefore we monitored the conversion of CCA to 7-OH-3-CCA by HPLC. HPLC analysis of the CCA reaction ([CCA] = 200 μM) revealed that unactivated RuP does not suppress ROS generation indicated by the presence of 85 ± 3 μM of CCA and 109 ± 3 μM of 7-OH-3-CCA for RuP1 and 73 ± 5 μM of CCA and 118 ± 2 μM of 7-OH-3-CCA for RuP2. Importantly, in the presence of the photoactivated RuP, no 7-OH-3-CCA is observed, with only unreacted CCA detected by HPLC, indicating that ligand release is required to effectively suppress this form of Cu-catalyzed ROS generation (Fig. 10B, S25B, S26–S32, Tables S5 and S6). The HPLC analysis agrees with what is observed by fluorescence measurements with the respective ligands, 6,6′-dmb (RuP1) and 2,9-dmp (RuP2). Together these results highlight that photoactivated ligand release from both RuP1 and RuP2 complexes can provide additional benefit *via* suppressing Cu-Aβ redox cycling and ROS generation.

## Summary

This study demonstrates the potential of photoactivatable RuP as dual-action modulators of Aβ aggregation and Cu-induced oxidative stress, addressing two central hallmarks of Alzheimer's disease. Upon light activation, RuP complexes release sterically encumbered bidentate ligands, unmasking exchangeable coordination sites on the Ru center for binding to the Aβ peptide, and thereby modulating its aggregation pathway. RuP photoactivation results in the rapid and consistent formation of amorphous aggregates across all stages of Aβ assembly (monomers, oligomers, and fibrils), as confirmed by turbidity, gel electrophoresis, and TEM. A BCA assay revealed that the amorphous aggregates have reduced solubility, and EDX analysis showed significant integration of the photoactivated RuP into these amorphous aggregates. Proteinase-K assays established that these aggregates have increased susceptibility to proteolytic degradation, suggesting improved clearance when compared to more structured β-sheet fibrils. Concurrently, the photo-released ligands (6,6′-dmb, 2,9-dmp) inhibit Cu-associated ROS generation. ESI-MS confirmed that the ligands can compete with the Aβ peptide for Cu ions, while an ascorbate oxidation assay demonstrated that the ligands inhibit Cu^II^/Cu^I^ redox cycling. Further, a CCA assay showed that the photoactivated RuP, and photoejected ligands, halt the production of hydroxyl radicals. Taken together, these results reveal that photoactivation of RuP simultaneously redirects Aβ aggregation toward amorphous, off-pathway aggregates and inhibits Cu-mediated ROS generation. While these findings underscore the promise of RuP, it should be noted that competition from abundant serum proteins (*e.g.*, albumin) could influence binding profiles *in vivo*, and this consideration will be important for future translation. Overall, the dual activity shown herein highlights the potential of RuP as versatile phototherapeutic agents for managing protein aggregation and metal-induced oxidative stress in neurodegenerative diseases. In the future, we intend to expand this approach to other aggregation-prone proteins, aiming for a broad, multifaceted strategy against protein aggregation pathologies.

## Author contributions

GL, SAM, and TS designed the research project. GL completed the experiments except for docking studies performed by ALA and density functional theory optimizations completed by SM. PSB and ROH synthesized RuP1 and RuP2. GL, SAM and TS analysed the data and wrote the manuscript.

## Conflicts of interest

There are no conflicts to declare.

## Supplementary Material

SC-OLF-D5SC05593H-s001

## Data Availability

All data associated with this manuscript is either available in the main file or in the supplementary information (SI). Supplementary information is available. See DOI: https://doi.org/10.1039/d5sc05593h.
