# Supplementary material for: Photoactivatable Ru(ii) polypyridyl complexes as dual action modulators of amyloid-beta peptide aggregation and Cu redox cycling
Source: Chem Sci. 2025 Oct 1;16(44):20914–23. doi: 10.1039/d5sc05593h (PMC12509088; doi:10.1039/d5sc05593h)
Supplement: SC-016-D5SC05593H-s001 [file SC-016-D5SC05593H-s001.pdf]

Supplementary Information (SI) for Chemical Science.
This journal is © The Royal Society of Chemistry 2025

SUPPORTING INFORMATION

Photoactivable Ru(II) Polypyridyl Complexes as Dual Action Modulators of Amyloid-Beta Aggregation and Cu Redox Cycling

Grace Leech[a], Alfredo Lopez Acosta[a], Samyadeb Mahato[a], Patrick C. Barrett[b], Rachel O. Hodges[b], Sherri A. McFarland[c*], and Tim Storr[a*]

[a]Department of Chemistry, Simon Fraser University, BC, Canda, V5A-1S6

[b]Department of Chemistry and Biochemistry, The University of North Carolina at Greensboro, Greensboro, NC, USA

[c]Department of Chemistry and Biochemistry, The University of Texas at Arlington, Arlington, Texas, USA, 76019

Experimental Information:

RuP1 and **RuP2** were synthesized as reported.[1,2] $A\beta_{1-16}$, and $A\beta_{1-42}$ were purchased from ChinaPeptides (Pudong New Area, SH, CN). All peptide was monomerized prior to use using Hexafluoro-2-propanol (HFIP) and further dissolved in DMSO following reported procedures.[3] The concentration of the stock peptide solution was determined by measuring the absorbance using a Thermo Nicolet UV nanodrop of Tyr^{10}, considered as free tyrosine, with an extinction coefficient of 1410 M^{-1} cm^{-1} at 280 nm. Photo-ejection experiments and ascorbate consumption assays were measured by UV-Vis on a Cary 5000 Spectrophotometer. Turbidity, BCA and CCA assays were conducted on a Synergy 4 MultiDetection microplate reader (BioTek). ESI-MS experiments were performed on a Bruker maXis Impact Quadrupole Time-of-Flight LC/MS system. High-Performance Liquid Chromatography (HPLC) was conducted using an Agilent 1100 series system equipped with an autosampler and an automated fraction collector. The stationary phase consisted of a Zorbax 5 µm C18 100 Å column (50 × 4.6 mm, analytical) with a flow rate of 0.6 mL/min. The analytical mobile phase A comprised 10 % acetonitrile in ultrapure water (18 MΩ, MilliQ A10) with 20 mM ammonium acetate and 20 µM EDTA. Mobile phase B consisted of 80 % acetonitrile and 10 % methanol in ultrapure water (18 MΩ, MilliQ A10), also containing 20 mM ammonium acetate and 20 µM EDTA. TEM, STEM and EDX were obtained using an OSIRIS FEI scanning TEM (STEM) operating at 200 kV. Emission spectra determination of the binding

affinities for each complex was performed using an Edinburgh Instruments FS5 spectrofluorometer equipped with a 150W Xenon arc lamp.

1.1.1 Photo-ejection Experiments

Ru(II) complexes were dissolved in 90% ddH_2O 10% DMSO and added to phosphate buffered saline (PBS, 0.01 M Na_2HPO_4 0.01 M KH_2PO_4, 0.14 M NaCl, 0.003 M KCl, pH 7.4). Photo-ejection experiments were conducted *via* UV-Vis, using a visible light source with a cool white colour (6000-6400 K) (SOLLA 30W LED, **Figure S1**). The sample and light source were kept at a constant distance of 10 cm for the photoactivation experiments. Data were collected from 200-820 nm. Irradiation intervals began every minute up until 15 minutes, after which data was collected every 5 minutes until 60 minutes. The photo-ejection time was determined when there were no further observed spectral changes. The photo-ejection kinetics were studied by plotting the normalized change in absorption where the greatest change had occurred throughout the course of the experiment, against irradiation time using previously reported methods.[4,5,6,7]

1.1.2 ESI Mass Spectrometry of Binding of Aβ Peptide to Ru(II) Complexes and Cu coordination complexes

Mass spectrometric analysis was performed using a Bruker maXis Impact Quadrupole Time-of-Flight LC/MS system, composed of an Agilent 1200 HPLC for sample introduction and a Bruker maXis Impact Ultra-High Resolution tandem TOF (UHR-Qq-TOF) mass spectrometer. Sample introduction was achieved *via* flow injection using the HPLC with a mobile phase consisting of 1:1 acetonitrile:water (0.1% formic acid). The system operated in positive electrospray ionization mode (+ESI) with the following settings: gas temperature of 200°C, gas flow of 8 L/min, nebulizer pressure of 2 bar, and capillary voltage set at 4500 V. Sample introduction was achieved *via* flow injection using the HPLC with a mobile phase consisting of 1:1 acetonitrile:water (0.1% formic acid).Data acquisition and processing were conducted using Compass 1.5 software. Samples were prepared as 200 μM of total protein ($A\beta_{1\text{-}16}$) in ammonium carbonate (0.01 M, pH 9) buffer with 0 or 1 eq. of Ru(II) complexes. Cu samples were prepared as 100 μM of total protein ($A\beta_{1\text{-}16}$) in ammonium carbonate (0.01 M, pH 9) buffer with 200 μM **RuP** and 100 μM of $CuCl_2$.

1.1.3 Light Scattering Assay

A light scattering assay was completed in filtered phosphate-buffered saline (0.01 M, pH 7.4) and turbidity was monitored by observing the changes in absorbance at 600 nm using a Synergy 4 MultiDetection microplate reader (BioTek). Peptide concentration was verified using a Nanodrop spectrophotometer. Final DMSO concentrations were kept below 5% (v/v) to ensure biologically relevant conditions. A 0 h absorbance (600 nm) scan was taken immediately, and the plate was then set to agitate for 24 h at 37 ºC, at which time an endpoint scan was taken. The experiment was performed in quadruplicate measurements. Congo red (CR) was used as the positive control.

1.1.4 Gel Electrophoresis and Western Blotting

Lyophilized $A\beta_{1-42}$ was dissolved in DMSO to obtain a stock solution of a concentration of 400 μM. The $A\beta_{1-42}$ stock solution was further diluted to 25 μM in PBS (0.01 M, pH 7.4). This was incubated at 37 ºC with continuous agitation to form aggregates in absence and presence of both non-activated and photoactivated Ru(II) complexes. Aliquots were collected at time points of 0 h and 24 h and frozen at -80 ºC until use. $A\beta_{1-42}$ was otherwise incubated at 4 ºC with continuous agitation overnight to form oligomeric species,[8] then Ru(II) complexes were added and photoactivated. Electrophoresis separation of peptide aggregates was performed using 8-16% Mini-PROTEAN® TGX Precast Gels (Bio-Rad) at 100 V for 80 minutes in running buffer (25 mM Tris, 192 mM glycine, 0.1% SDS). The gels were then transferred to a nitrocellulose membrane for 1 h at 100 V on ice, followed by blocking of the membrane in 3 % BSA in Tris-buffered saline with Tween® (TBS-T) (0.02 M Tris, 0.15 M NaCl, 0.003 M KCl, 0.5 % Tween) for 1 h at room temperature. The membrane was then incubated in a solution with primary antibody, 6E10 (Biolegends) (1:2000 dilution), that recognizes $A\beta_{1-16}$, for 2 h at room temperature. After washing the membrane 3x10 min with TBS-T, the membrane was then incubated in secondary antibody, Horseradish peroxidase (Caymen Chemicals) (1:10,000) for 1h at room temperature. The membrane was then washed 3x10 min with TBS-T and developed using a Thermo Scientific SuperSignal® West Pico Chemiluminescent Substrate kit to visualize the Aβ species using a Bio-Rad ChemiDoc™ MP imaging system.

1.1.5 Transmission Electron Microscopy (TEM) and Electron Dispersive X-ray Spectroscopy

TEM samples were prepared from the 1:1 Ru(II)/Aβ samples from the Western blot assay at incubation time points of 0 h for the monomeric form and 96 h at 37 ºC for fibrillar aggregates of $A\beta_{1-42}$. TEM grids were prepared using previously reported methods.[9,10] Drops of samples (5 μL) of sample was placed onto the grid and allowed to sit for 3 minutes, followed by washing with water droplets on a sheet of parafilm. The grid was then placed on top of a Uranyless stain (Electron Microscopy Sciences) for 1 minute. The grid was allowed to air-dry for 15 minutes prior to imaging. Bright field images were obtained using a FEI Technai Osiris STEM at 200 kV. High-angle annular dark-field (HAADF) STEM images were then obtained to use for elemental mapping *via* Energy dispersive X-ray Spectroscopy (EDX) of Ru (Ru L_{α}). All images were taken at a consistent scale bar of 200 nm for comparison purposes.

1.1.6 Bicinchoninic acid (BCA) Assay

$A\beta_{1-42}$ stock solution (400 μM) was diluted in PBS buffer (0.01 M, pH 7.4) to a final concentration of 60 μM in the absence and presence of Ru(II) complexes (1 equiv.) both unactivated and photoactivated for a period of 24 h. The samples were first centrifuged at 14,000 g for 5 minutes and aliquots were taken at a 0 h time point and frozen at -80 ºC until use. The solution was then added to a 96 well plate (20 μL) in triplicate measurements for each time point, and 200 μL of working reagent (Thermo Fisher BCA Protein Assay® kit) was added to each well. The plate was then incubated for 30 minutes at 37 ºC, and the solubility of the peptide was determined by its concentration in the supernatant, measuring the absorbance at 562 nm using a Synergy 4 MultiDetection microplate reader (BioTek).

1.1.7 Docking Studies

Molecular docking was performed with AutoDock Vina 1.2.5 relying on a stochastic approach combined with BFGS gradient-based optimizer for binding energy calculations.[13] Docking results were visualized using Discovery Studio 2024 to better understand the predicted binding sites and interactions. Iron is better parameterized in comparison to Ru in AutoDock and thus, both Fe(II) and Ru(II) compounds were first optimized by DFT (Gaussian 16 RevC.01 (B3LYP/LANL2DZ in PCM continuum solvent model(water))) and compared for structural differences. The structures of

the Ru and Fe complexes were found to be essentially identical based on structural optimization therefore the Fe complexes were used in the docking studies.

AutoDockTools 1.5.7 was used to obtain the required pdbqt files of metal complexes and peptide aggregates. To prepare each geometrically optimized complex for docking, Gasteiger charges were calculated without merging non-polar hydrogens and maintaining the central metal atom (II) charge. Two $A\beta_{1-42}$ structures were used for docking, PDB: 5OQV[14] and PBD: 2MXU[15] obtained from cryo-electron microscopy and solid state NMR techniques, respectively. Kollman charges were calculated for both target structures without merging non-polar hydrogens to prepare for blind docking. The configuration file was set to an energy range of 4 and exhaustiveness of 20, which afforded the 9 most stable binding poses for each complex. Results were ranked based on their respective binding energies.

1.1.8 Proteinase-K Assay

$A\beta_{1-42}$ alone and in the presence of 1 equiv. of photoactivated **RuP** in PBS buffer (0.01 M, pH 7.4) was allowed to aggregate for 96 h at 37 ºC. Then 2.5 uL of each sample was dotted onto a nitrocellulose membrane in triplicate measurements, with and without 0.2 equiv. of proteinase-k (PK). The membrane was allowed to dry for 10 minutes. Blocking of the membrane was performed in 3 % BSA in Tris-buffered saline with Tween® (TBS-T) (0.02 M Tris, 0.15 M NaCl, 0.003 M KCl, 0.5 % Tween) for 1 h at room temperature. The membrane was then incubated in a solution with primary antibody, 6E10 (Biolegends) (1:2000 dilution), that recognizes $A\beta_{1-16}$, for 2 h at room temperature. After washing the membrane 3 x 10 minutes with TBS-T, the membrane was then incubated in secondary antibody, Horseradish peroxidase (Caymen Chemicals) (1:10,000) for 1 h at room temperature. The membrane was then washed 3 x 10 min with TBS-T and developed using a Thermo Scientific SuperSignal® West Pico Chemiluminescent Substrate kit to visualize the Aβ species using a Bio-Rad ChemiDoc™ MP imaging system. *ImageJ* software was used to obtain the integrated density of the signals observed on the nitrocellulose membrane.

1.1.9 Ascorbate Consumption Assay

Ascorbate oxidation/consumption was monitored spectrophotometrically by measuring its absorbance at 265 nm at room temperature in a quartz cuvette in PBS buffer (0.01 M, pH 7.4). Reaction mixtures contained 100 μM of ascorbate, 25 μM $CuCl_2$ or 25 μM Cu-$A\beta_{1-16}$ and 12.5-50

μM 6,6’-dimethyl-2,2’-bipyridyl (6,6′-dmb) or 2,9-dimethyl-1,10-phenanthroline (2,9-dmp). A control of ascorbate alone was monitored over 15 minutes to ensure stability under our conditions. The absorbance at 265 nm was measured every 10 seconds for a total of 15 minutes. Cu-Aβ_{1-16} was added at 1 min and the respective ligands (6,6’-dmb or 2,9-dmp) were added at the 3 min timepoint. Ru complexes were not used in this assay due to absorption interference.

1.1.10 Coumarin-3-carboxylic acid (CCA) Assay and HPLC Analysis

Coumarin-3-carboxylic acid (3-CCA) was used to detect hydroxyl radicals (·OH) produced by Cu and Cu-Aβ_{1-16} in the presence of L-ascorbic acid using a Synergy 4 MultiDetection microplate reader (BioTek). The CCA assay was conducted in triplicate in a flat-bottomed black 96-well plate (Microtest, BD Falcon). CCA was dissolved in PBS. $CuCl_2$ (25 μM), CCA (100 μM), L-ascorbic acid (200 μM) Aβ_{1-16} (25 μM) 6,6’-dmb (50 μM) or 2,9-dmp (50 μM) were the final reagent concentrations. Reaction of CCA with ·OH produces 7-OH-CCA, a fluorescent compound with λ_{ex} = 390 nm and λ_{em} = 450 nm. The reaction was monitored at room temperature for 60 min, scanning every minute. Ru complexes were not monitored in this manner due to absorption overlap. In order to determine the ability of the **RuP** complexes (both unactivated and activated) for their ability to limit ROS generation in the CCA assay, the relative concentrations of 3-CCA and 7-OH-3-CCA were quantified by HPLC. Calibration curves of each commercially available pure product were generated using four different concentrations (50-200 μM in 1 mL PBS). The integrated peak areas were plotted against their known concentrations to assess linearity. Once a linear correlation was established, product yields from the reaction mixtures were quantified by correlating the measured peak areas with the calibration curve. See **Figures S24-30** for further details.

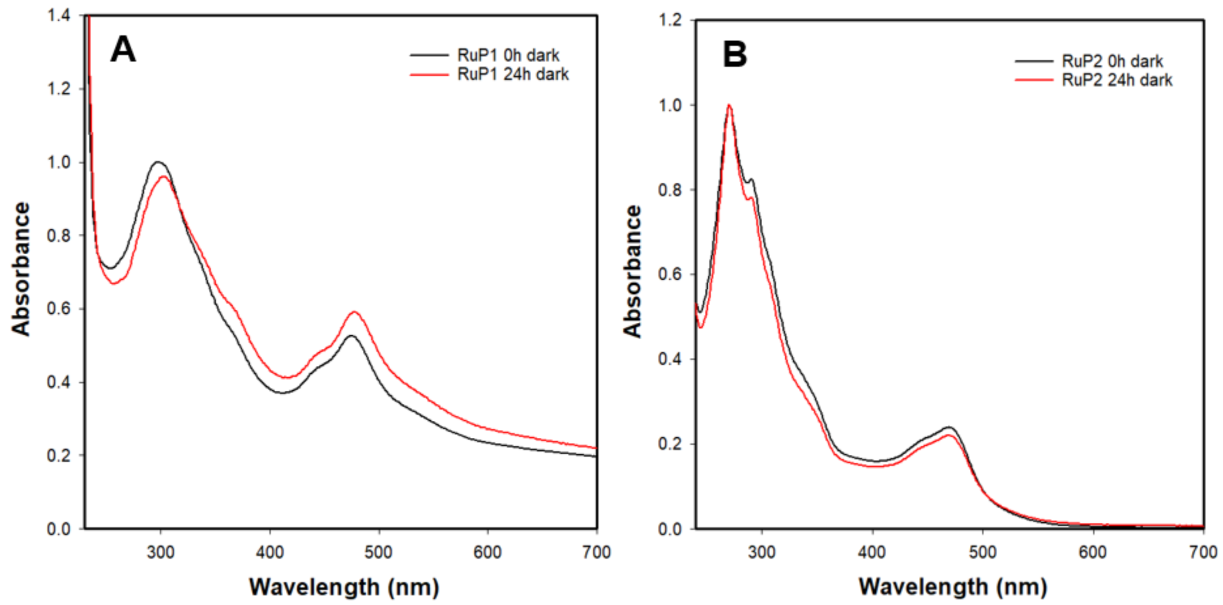

Figure S1. UV-Vis Spectra of unactivated **RuP1** and **RuP2** at 0 h and 24 h. Conditions: **RuP** = 25 µM in phosphate buffer saline (PBS, 0.01 M, pH 7.4).

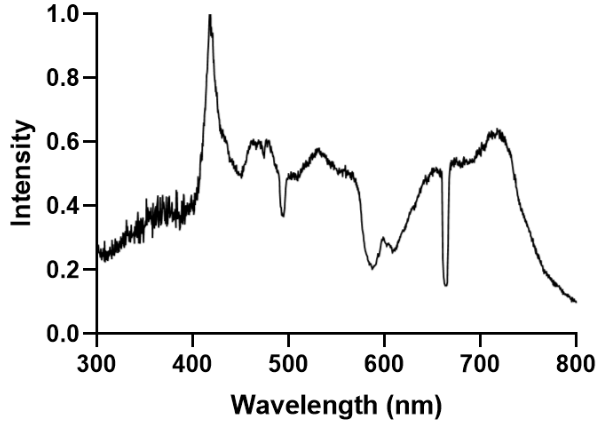

Figure S2. Reflection Scan of light source (SOLLA, LED 30W) affording maximal intensity at 420 nm.

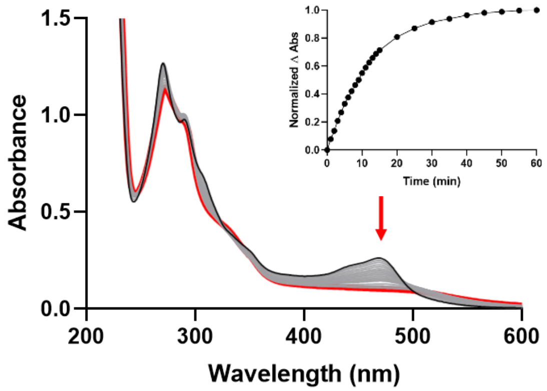

Figure S3. UV-Vis Absorption spectra of **RuP2** in PBS (0.01 M, pH 7.4). The unactivated sample (black) was photoactivated until an endpoint was reached (red), allowing for the determination of the photoactivation time required for the reaction to come to completion. Inset shows the change in absorbance at 471 nm (**RuP2**) over time with an endpoints at 35 min (100.17 J/cm^2).

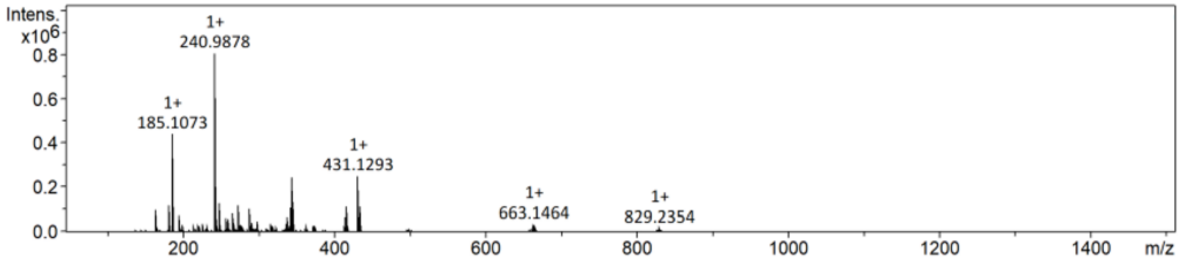

Figure S4. ESI-MS of photoactivated **RuP1** in NH_4CO_3 buffer (20 mM, pH 9.0) showing evidence of selective ligand ejection of 6,6'-dmb (m/z = 185.10 (1+)) affording readily exchangeable coordination sites for Aβ peptide binding and a **RuP1**OH adduct (m/z = 663.14 (1+)) where OH has occupied one of the coordination sites made available due to ligand dissociation. The complex remains partially intact, represented at m/z = 829.23 (1+).

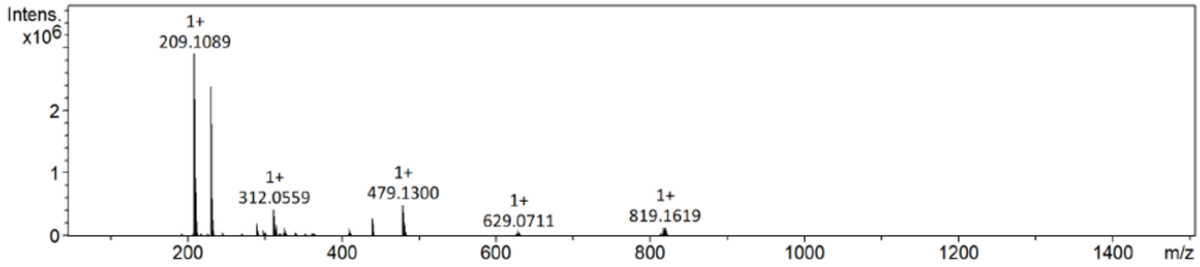

Figure S5. ESI-MS of photoactivated **RuP2** in NH_4CO_3 buffer (20 mM, pH 9.0) showing evidence of selective ligand ejection of 2,9'-dmp (m/z = 209.10 (1+)) affording readily exchangeable coordination sites for Aβ peptide binding and a **RuP2**OH adduct (m/z = 629.07 (1+)) where OH has occupied one of the coordination sites made available due to ligand dissociation. The complex remains partially intact, represented at m/z = 819.16 (1+).

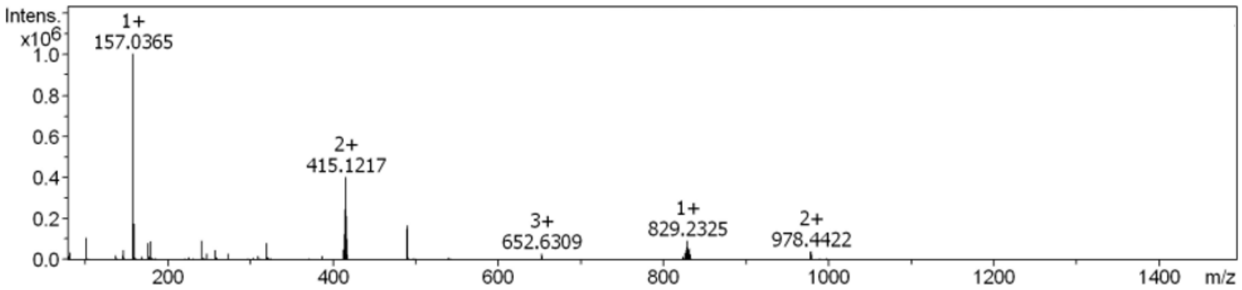

Figure S6. ESI-MS of unactivated **RuP1** in the presence of Aβ$_{1-16}$ in NH_4CO_3 buffer (20 mM, pH 9.0) showing no evidence of adduct formation. Peaks observed are reflective of Aβ$_{1-16}$ (m/z = 978.44 (2+), 652.63 (3+) and **RuP1** (m/z = 829.23 (1+), 415.12 (2+)).

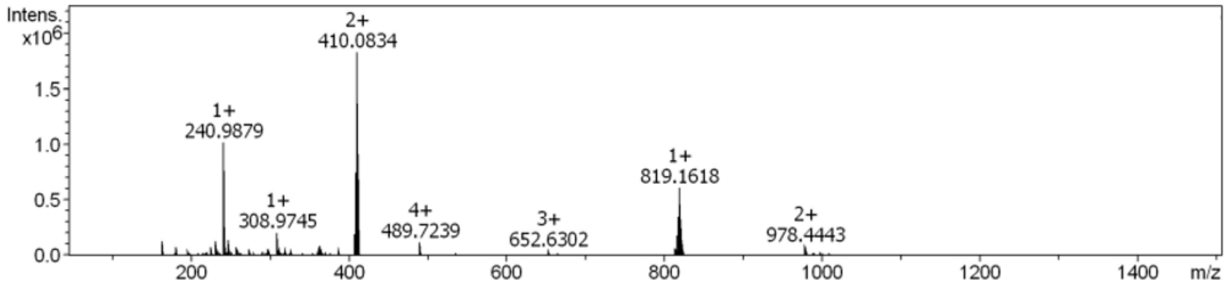

Figure S7. ESI-MS of unactivated **RuP2** in the presence of 1 equiv. of Aβ$_{1-16}$ in NH_4CO_3 buffer (20 mM, pH 9.0) showing no evidence of adduct formation. Peaks observed are reflective of Aβ$_{1-16}$ (m/z = 978.44 (2+), 652.63 (3+), 489.72 (4+)) and **RuP2** (m/z = 819.16 (1+), 410.108 (2+)).

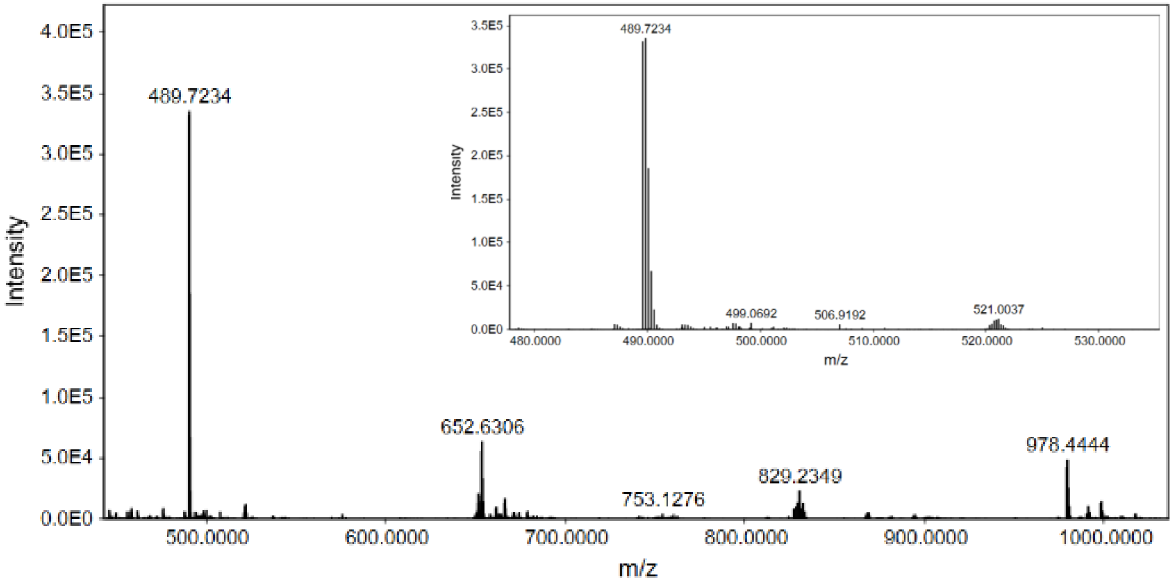

Figure S8. ESI-MS of photoactivated **RuP1** in the presence of 1 equiv. of Aβ$_{1-16}$ in NH_4CO_3 buffer (20 mM, pH 9.0) showing evidence of **RuP1**-Aβ$_{1-16}$ adduct formation (m/z = 521.00 (5+)). Peaks at m/z = 489.72 (4+), 652.63 (3+) and 978.44 (2+) are all reflective of Aβ$_{1-16}$. Inset shows region of interest highlighting evidence of **RuP1**-Aβ$_{1-16}$ adduct formation (m/z = 521.00 (5+)).

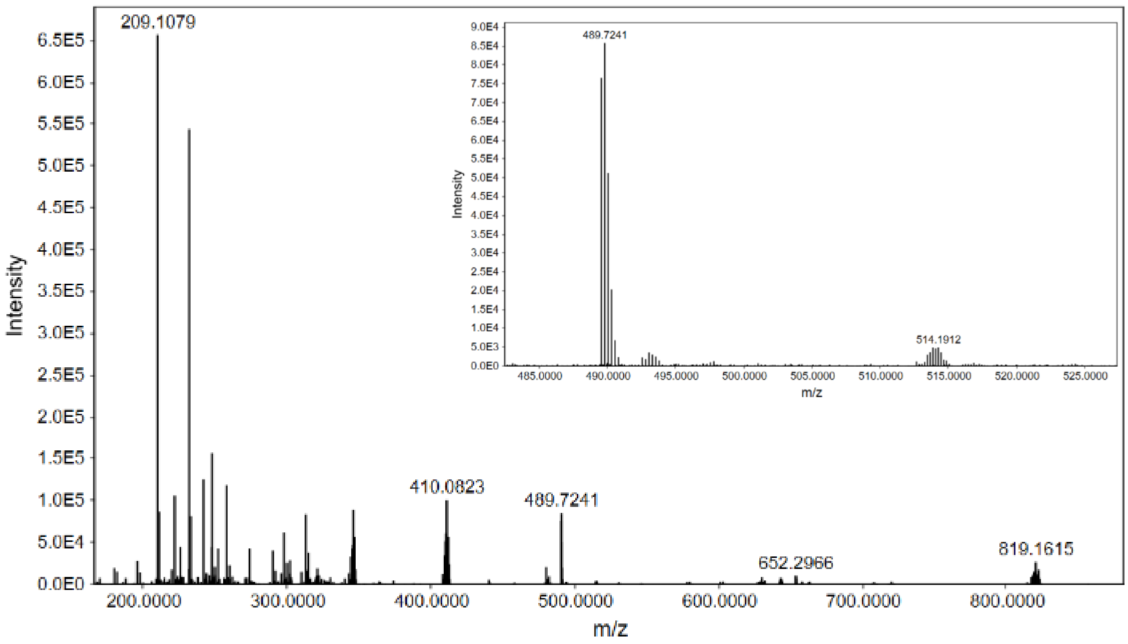

Figure S9. ESI-MS of photoactivated **RuP2** in the presence of A$\beta_{1\text{-}16}$ in NH_4CO_3 buffer (20 mM, pH 9.0) showing evidence of **RuP2**-A$\beta_{1\text{-}16}$ adduct formation (m/z = 514.19 (5+)). Peaks at m/z = 489.72 (4+) and 652.29 (3+) are reflective of A$\beta_{1\text{-}16}$. Peak at m/z = 209.11 (1+) is reflective of free 2,9'-dmp ligand. Peaks at m/z = 819.16 (1+) and 410.08 (2+) are reflective of intact **RuP2** complex. Inset shows region of interest highlighting evidence of **RuP2**-A$\beta_{1\text{-}16}$ adduct formation (m/z = 514.19 (5+)).

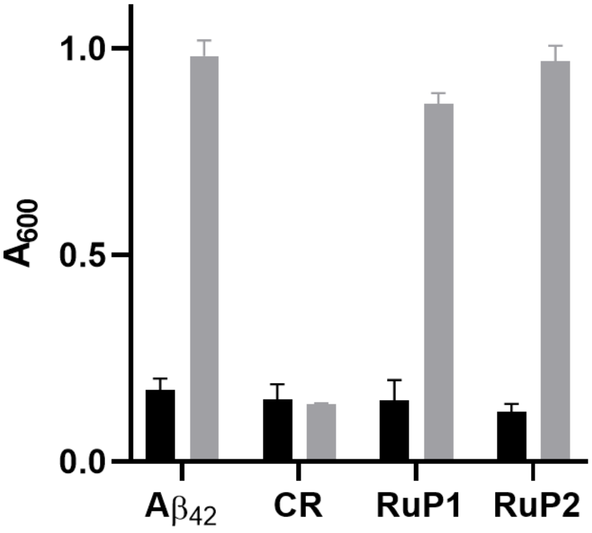

Figure S10. Light scattering turbidity assay of 25 μM A$\beta_{1\text{-}42}$ and 1 equiv. **RuP** in PBS (0.01 M, pH 7.4) at incubation times of 0 h (black) and 24 h (grey) unactivated, CR = Congo Red.

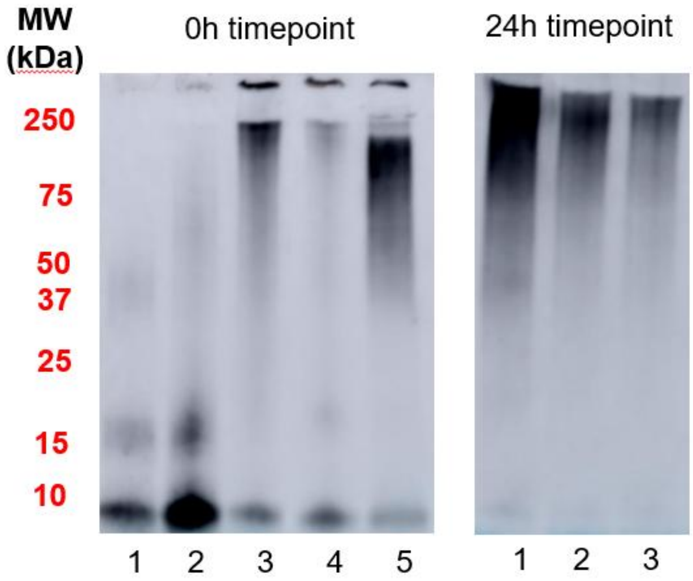

Figure S11. Western blot of 25 μM A$\beta_{1\text{-}42}$ in the absence and presence of 1 equiv. **RuP** in PBS (0.01 M, pH 7.4) at incubation times 0 h and 24 h. 0 h timepoint: lane 1: A$\beta_{1\text{-}42}$, lane 2: Photolyzed **RuP1** + A$\beta_{1\text{-}42}$, lane 3: **RuP1** + A$\beta_{1\text{-}42}$ photoactivated, lane 4: Photolyzed **RuP2** + A$\beta_{1\text{-}42}$, lane 5: **RuP2** + A$\beta_{1\text{-}42}$. 24 h timepoint: lane 1: A$\beta_{1\text{-}42}$, lane 2: Photolyzed **RuP1** + A$\beta_{1\text{-}42}$, lane 3: Photolyzed **RuP2** + A$\beta_{1\text{-}42}$.

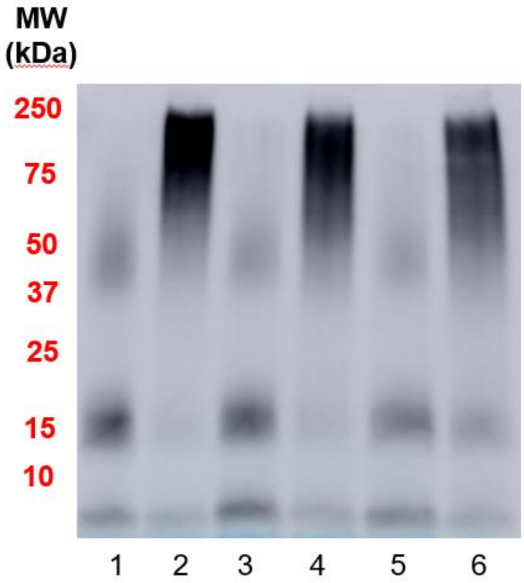

Figure S12. Western Blot of A$\beta_{1\text{-}42}$ alone (25 μM) and in the presence of 2 equiv. 6,6'-dmb or 2,9-dmp ligands. Lane 1: A$\beta_{1\text{-}42}$ 0 h, Lane 2: A$\beta_{1\text{-}42}$ 24 h, Lane 3: A$\beta_{1\text{-}42}$ + 6,6'-dmb 0 h, Lane 4: A$\beta_{1\text{-}42}$ + 6,6'-dmb 24 h, Lane 5: A$\beta_{1\text{-}42}$ + 2,9-dmp 0 h, Lane 6: A$\beta_{1\text{-}42}$ + 2,9-dmp 24 h.

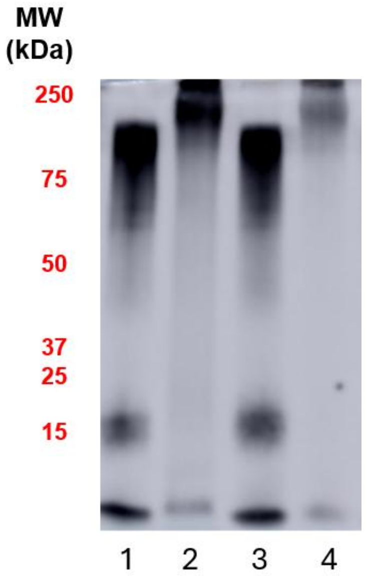

Figure S13. Western blot of 25 μM A$\beta_{1\text{-}42}$ oligomers in the presence of 1 equiv. of photoactivated **RuP** in PBS (0.01 M, pH 7.4) Lane 1: A$\beta_{1\text{-}42}$ oligomers, lane 2: + **RuP1** photoactivated, lane 3: A$\beta_{1\text{-}42}$ oligomers and lane 4: + **RuP2** photoactivated.

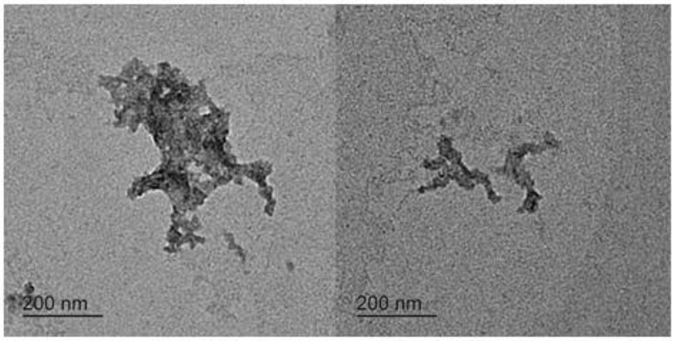

Figure S14. TEM images of A$\beta_{1\text{-}42}$ oligomers (25 μM) in the presence of 1 equiv. of photoactivated **RuP**1 (left) and **RuP2** (right) in PBS buffer (0.01 M, pH 7.4).

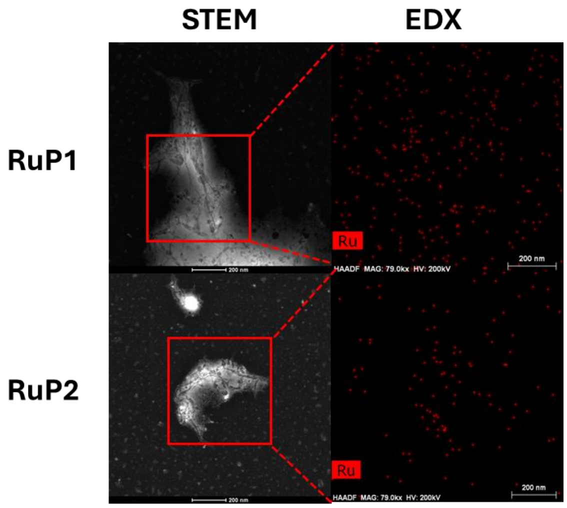

Figure S15. HAADF STEM & EDX of 25 μM A$\beta_{1\text{-}42}$ in the presence of 1 equiv. unactivated **RuP** after 96h aggregation. Red Box represents the area used for elemental mapping.

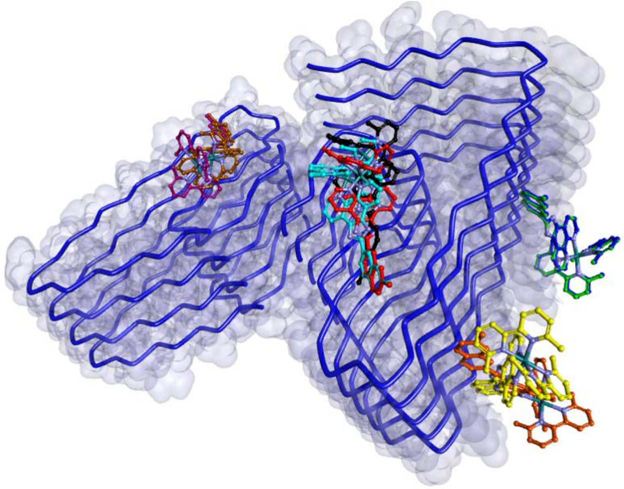

Figure S16. Potential binding sites of unactivated **RuP1** to PDB structure 5OQV. Different complex coloration was used to indicate different binding sites, where red is denoted as the most stable binding mode, however all calculated binding poses are of similar energy (**Table S1**).

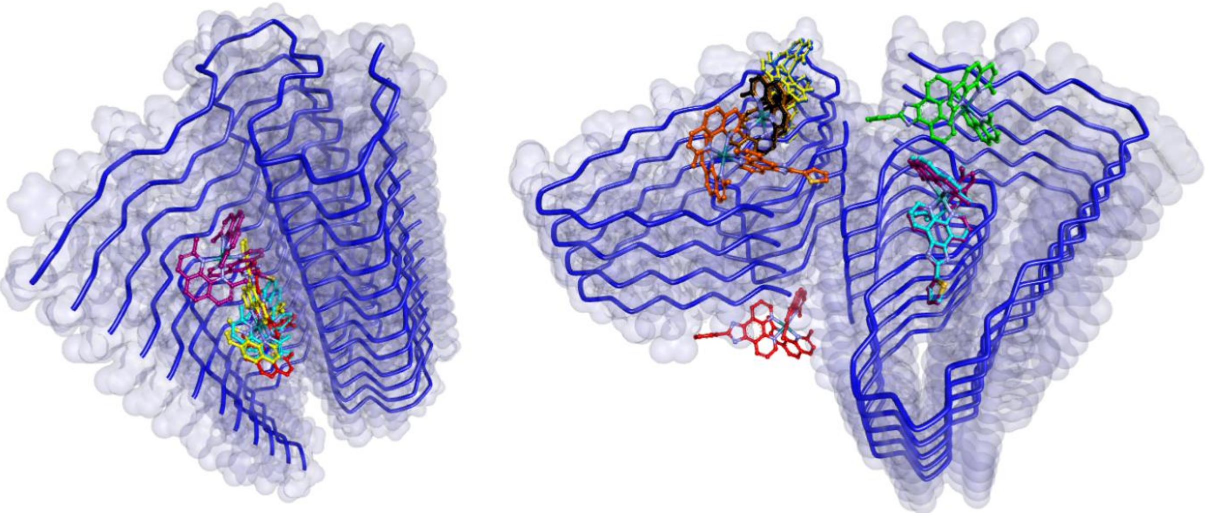

Figure S17. Potential binding sites of unactivated **RuP2** to PDB structures 5OQV (left) and 2MXU (right). Different complex coloration was used to indicate different binding sites, where red is denoted as the most stable binding mode, however all calculated binding poses are of similar energy (**Table S2, S4**).

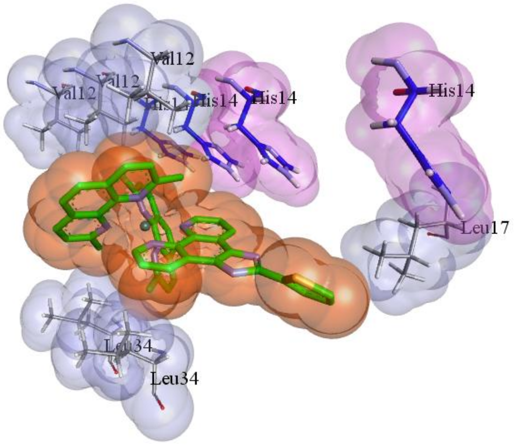

Figure S18. Molecular docking of the most stable binding mode of unactivated **RuP2** to PDB structure 2MXU identifying potential interactions with amino acids Leu^{17}, His^{14}, Val^{12} and Leu^{34}.

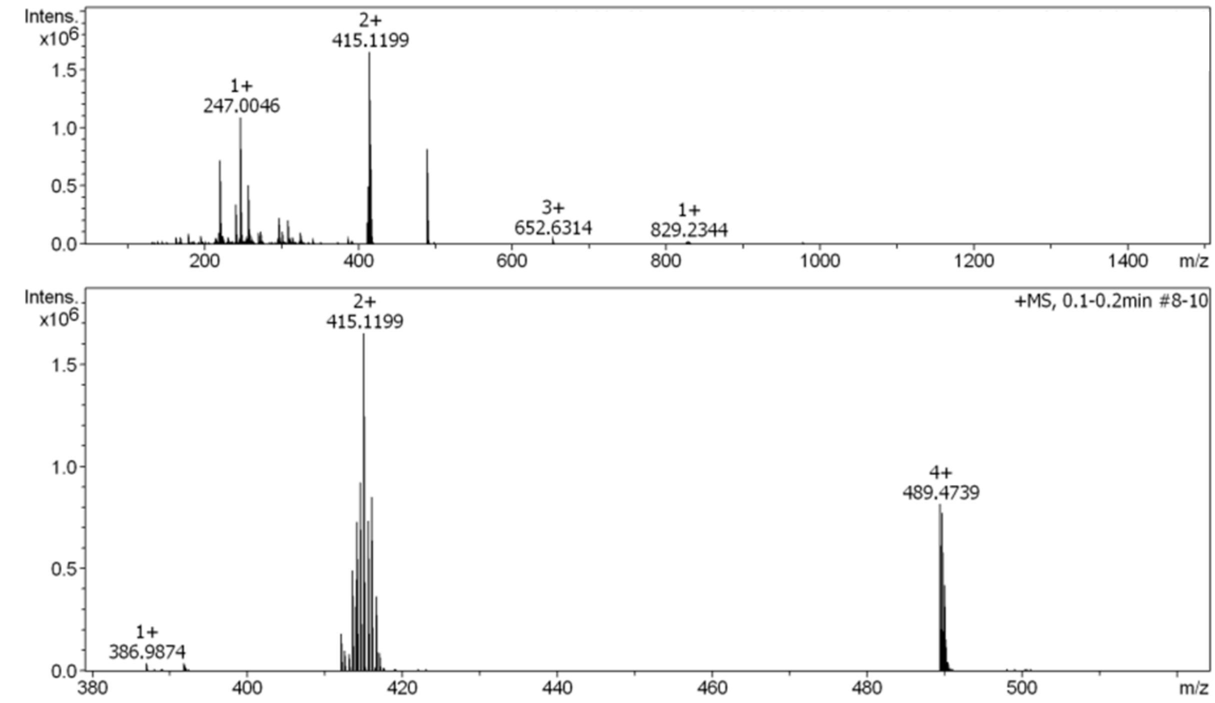

Figure S19. ESI-MS of unactivated **RuP1** + Cu-A$\beta_{1\text{-}16}$ in NH_4CO_3 buffer (20 mM, pH 9.0) showing evidence of intact **RuP1** (m/z = 415.12 (2+) and 829.23 (1+)) and A$\beta_{1\text{-}16}$ (m/z = 489.47 (4+) and 652.63 (3+)) and no evidence of Cu adduct ($Cu(bpy)_2{}^{1+}$) formation. Conditions: **RuP** = 200 μM, A$\beta_{1\text{-}16}$ = 100 μM, $CuCl_2$ = 100 μM.

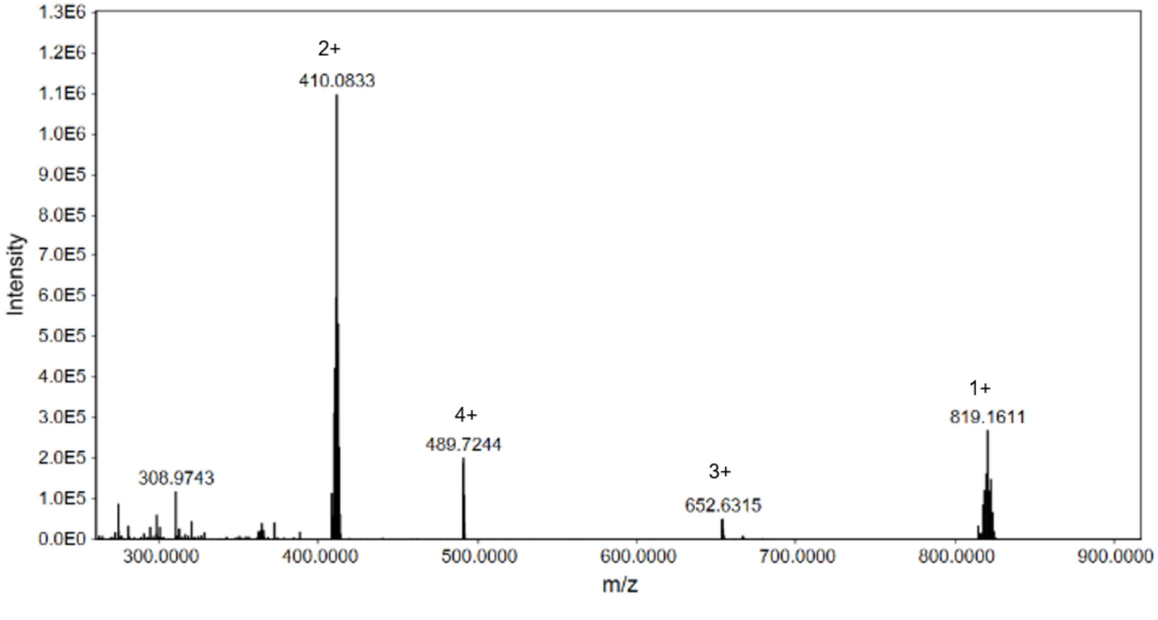

Figure S20. ESI-MS of unactivated **RuP2** + Cu-A$\beta_{1\text{-}16}$ in NH_4CO_3 buffer (20 mM, pH 9.0) showing evidence of intact **RuP2** (m/z = 410.08 (2+) and 819.16 (1+)), the A$\beta_{1\text{-}16}$ peptide (m/z = 489.72 (4+) and 652.63 (3+)) and no evidence of Cu adduct formation ($Cu(phen)_2{}^{1+}$) formation. Conditions: **RuP** = 200 μM, A$\beta_{1\text{-}16}$ = 100 μM, $CuCl_2$ = 100 μM.

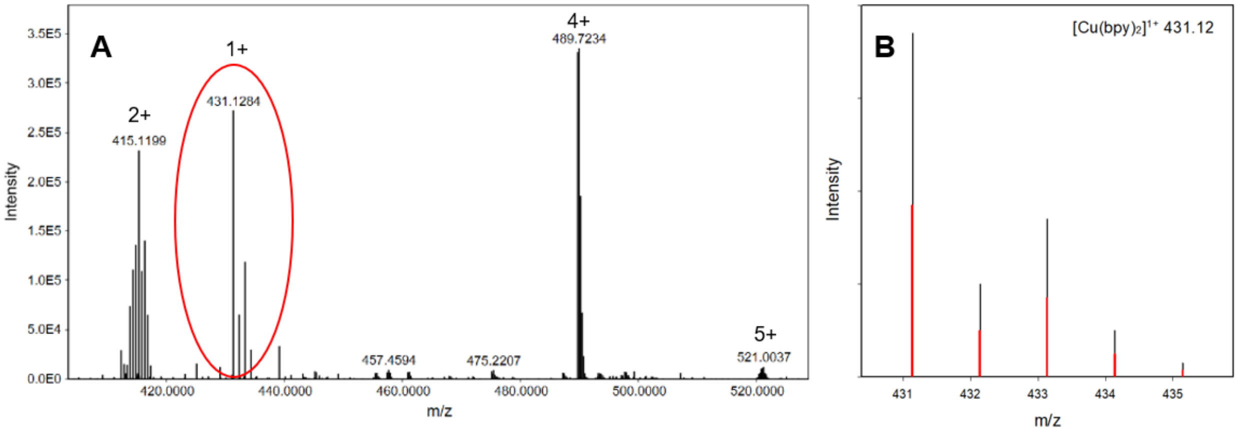

Figure S21. (A) ESI-MS of photoactivated **RuP1** + Cu-Aβ_{1-16} in NH_4CO_3 buffer (20 mM, pH 9.0) showing evidence of **RuP1** (m/z = 415.12 (2+), the Aβ_{1-16} peptide (m/z = 489.72 (4+)), the **RuP1**-Aβ_{1-16} adduct (m/z = 521.00 (5+)) and evidence of Cu adduct formation ($[Cu(bpy)_2]^{1+}$) at m/z = 431.14 (1+). (B) Inset showing ($[Cu(bpy)_2]^{1+}$) at m/z = 431.14 (1+). and the calculated isotopic pattern in red. Conditions: **RuP** = 200 µM, Aβ_{1-16} = 100 µM, $CuCl_2$ = 100 µM.

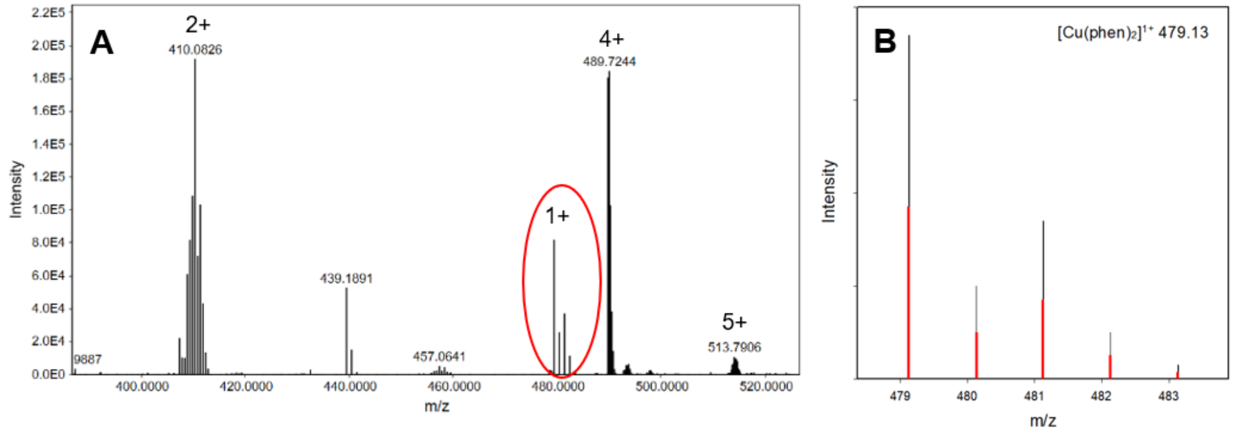

Figure S22. (A) ESI-MS of photoactivated $CuCl_2$RuP2Aβ_{16} in NH_4CO_3 buffer (20 mM, pH 9.0) showing evidence of **RuP2** (m/z = 410.08 (2+), the Aβ_{1-16} peptide (m/z = 489.72 (4+)), the **RuP2**-Aβ_{1-16} adduct (m/z = 513.79 (5+)) and evidence of Cu adduct formation ($Cu(phen)_2{}^{1+}$) at m/z = 479.13 (1+). (B) Inset showing ($[Cu(phen)_2]^{1+}$) at m/z = 479.13 (1+). and the calculated isotopic pattern in red. Conditions: **RuP** = 200 µM, Aβ_{1-16} = 100 µM, $CuCl_2$ = 100 µM.

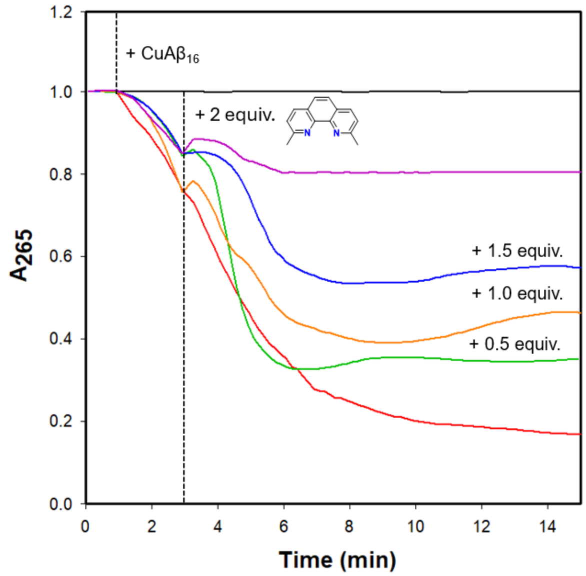

Figure S23. Ascorbate consumption assay measuring absorbance at 265 nm over time in PBS (0.01 M, pH 7.4). Ascorbate alone (black), and upon addition of Aβ_{1-16}-Cu (red) at the 1 min mark, followed by addition of 0.5 (green), 1.0 (orange), 1.5 (blue) and 2.0 (purple) equivalents 2,9-dmp at the 3 min mark. Conditions: [Asc] = 100 µM, [$CuCl_2$] = 25 µM, [2,9-dmp] = 12.5-50 µM, [Aβ_{1-16}] = 25 µM.

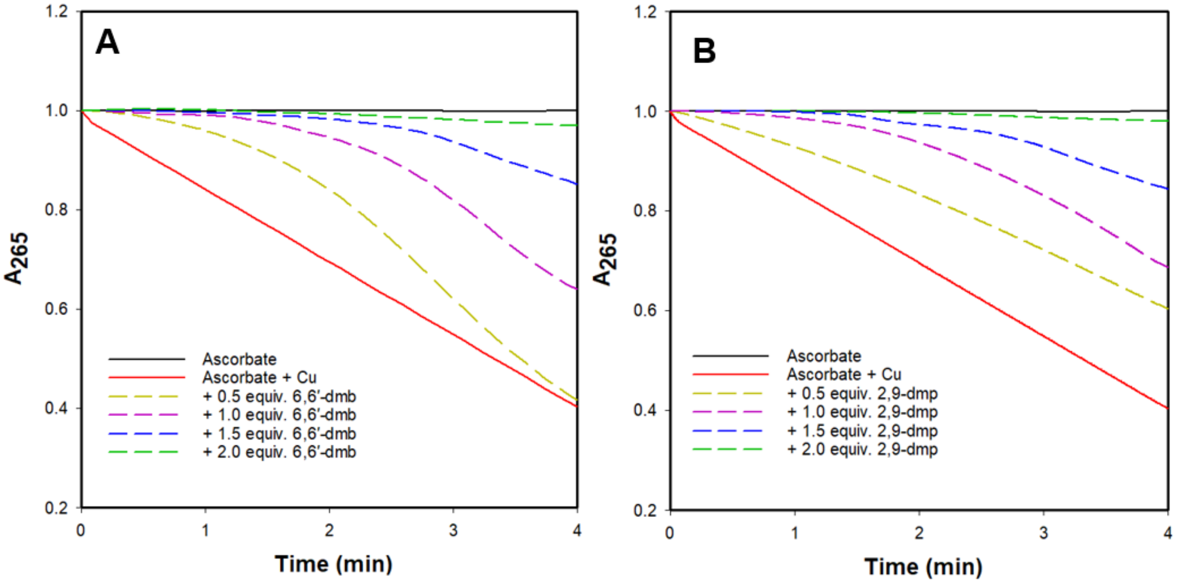

Figure S24. A) Ascorbate consumption assay measuring absorbance at 265 nm over 4 minutes in the absence or presence of Cu and various equivalencies of 6,6′-dmb and (B) 2,9-dmp in PBS (0.01 M, pH 7.4). Conditions: [Asc] = 100 µM, [$CuCl_2$] = 25 µM, [6,6′-dmb or 2,9-dmp] = 12.5-50 µM, [Aβ_{1-16}] = 25 µM.

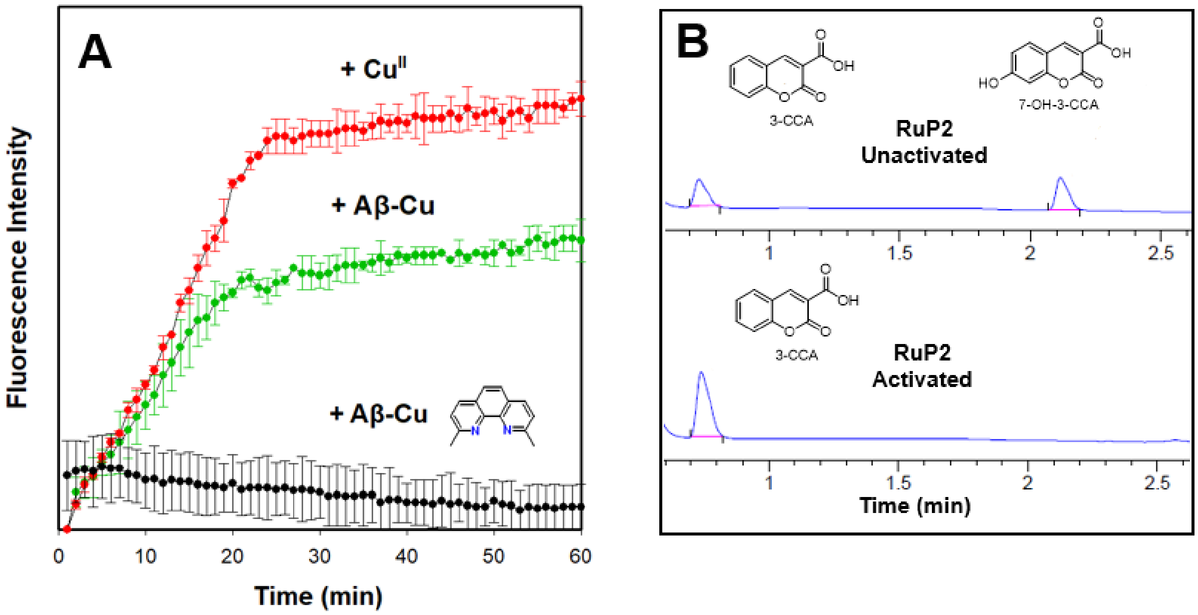

Figure S25. A) CCA assay for ·OH detection measured by fluorescence, λ_{ex} 390 nm and λ_{em} 450 nm. (B) HPLC of CCA assay with **RuP2** unactivated and activated in PBS (0.01 M, pH 7.4). Conditions: [Asc] = 200 µM, [CCA] = 200 µM, [$CuCl_2$] = 25 µM, [**RuP2** or 2,9-dmp] = 50 µM, [$A\beta_{1-16}$] = 25 µM.

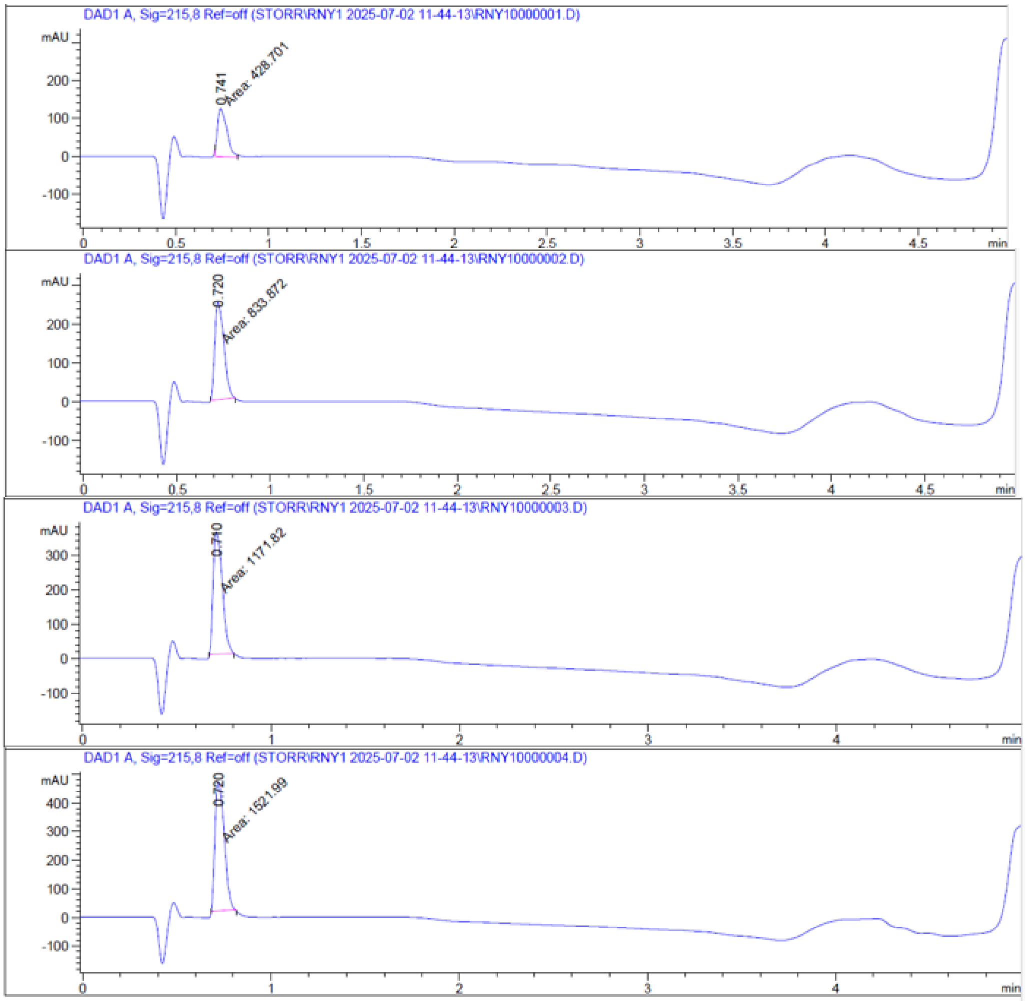

Figure S26. HPLC of 3-CCA at concentrations of 50 µM (top), 100 µM, 150 µM and 200 µM (bottom) in PBS (0.01 M, pH 7.4).

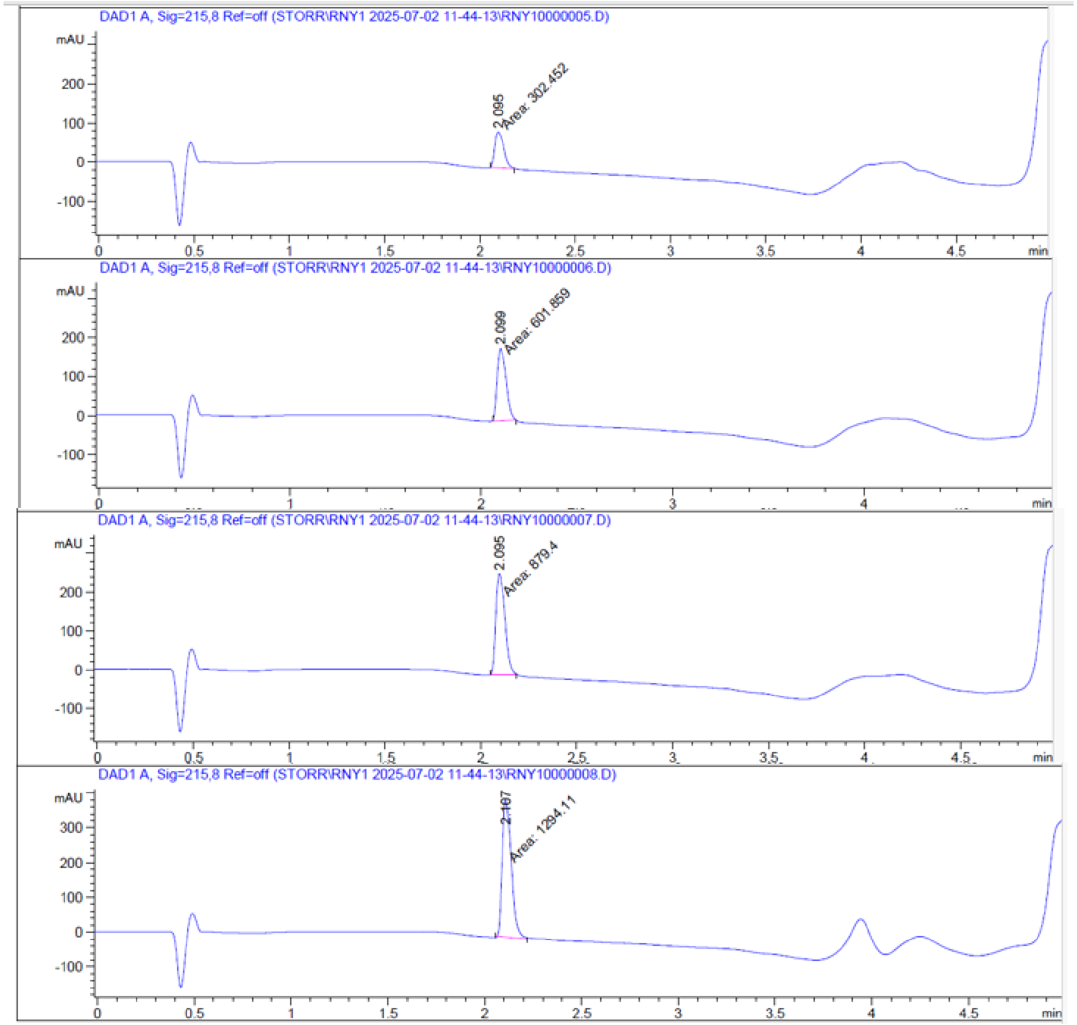

Figure S27. HPLC of 7-Hydroxy-3-CCA at concentrations of 50 µM (top), 100 µM, 150 µM and 200 µM (bottom) in PBS (0.01 M, pH 7.4).

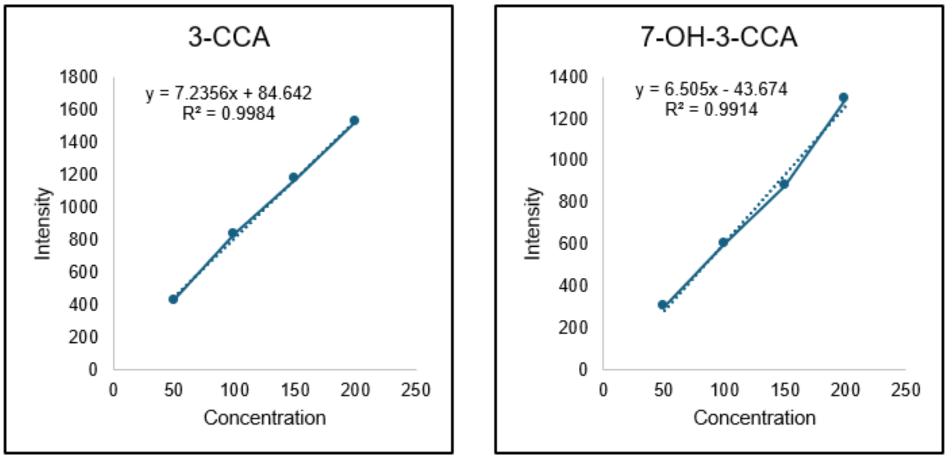

Figure S28. Calibration plots of HPLC standards, 3-CCA and 7-OH-3-CCA.

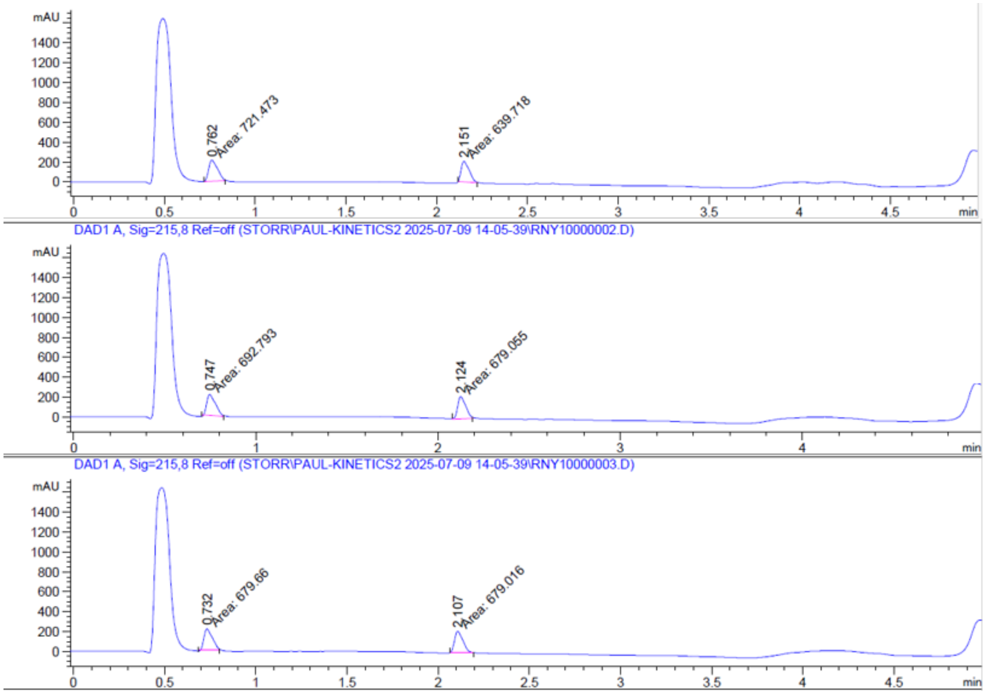

Figure S29. HPLC of **RuP1** unactivated measured in triplicate, in PBS (0.01 M, pH 7.4). Conditions: [Asc] = 200 μM, [CCA] = 200 μM, [$CuCl_2$] = 25 μM, [RuP1] = 50 μM, [A$\beta_{1\text{-}16}$] = 25 μM.

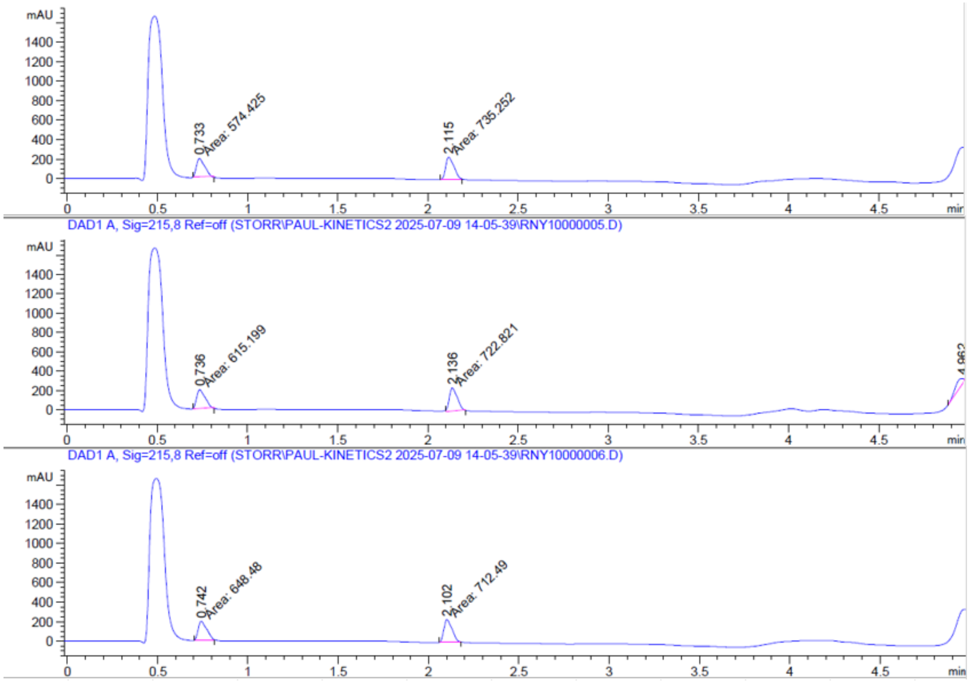

Figure S30. HPLC of **RuP2** unactivated measured in triplicate, in PBS (0.01 M, pH 7.4). Conditions: [Asc] = 200 μM, [CCA] = 200 μM, [$CuCl_2$] = 25 μM, [RuP2] = 50 μM, [A$\beta_{1\text{-}16}$] = 25 μM.

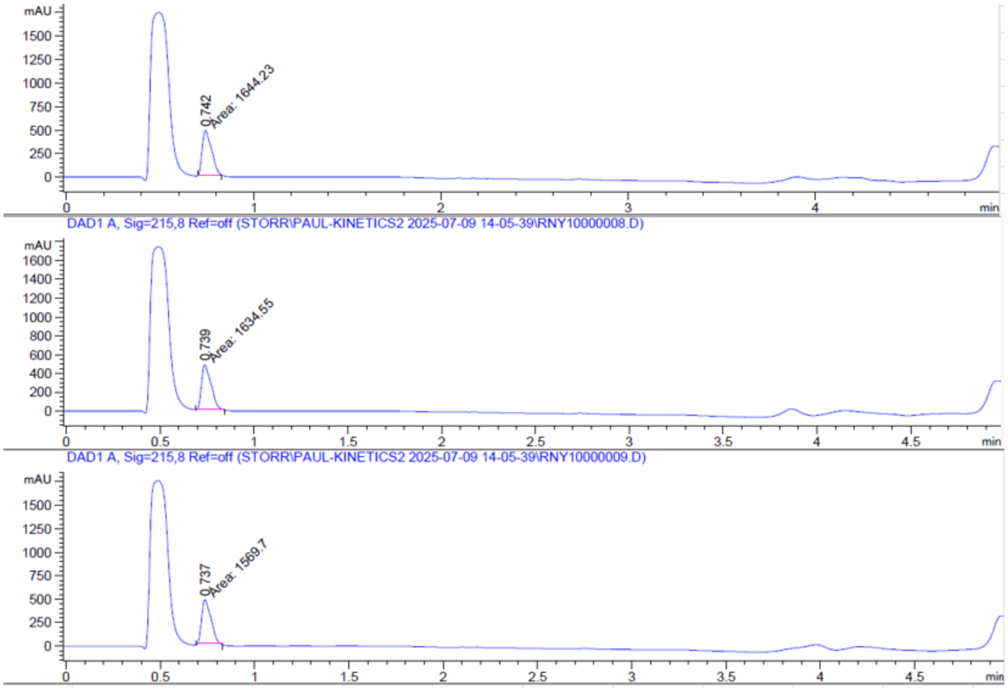

Figure S31. HPLC of **RuP1** photoactivated measured in triplicate, in PBS (0.01 M, pH 7.4). Conditions: [Asc] = 200 μM, [CCA] = 200 μM, [$CuCl_2$] = 25 μM, [RuP1] = 50 μM, [$A\beta_{1\text{-}16}$] = 25 μM.

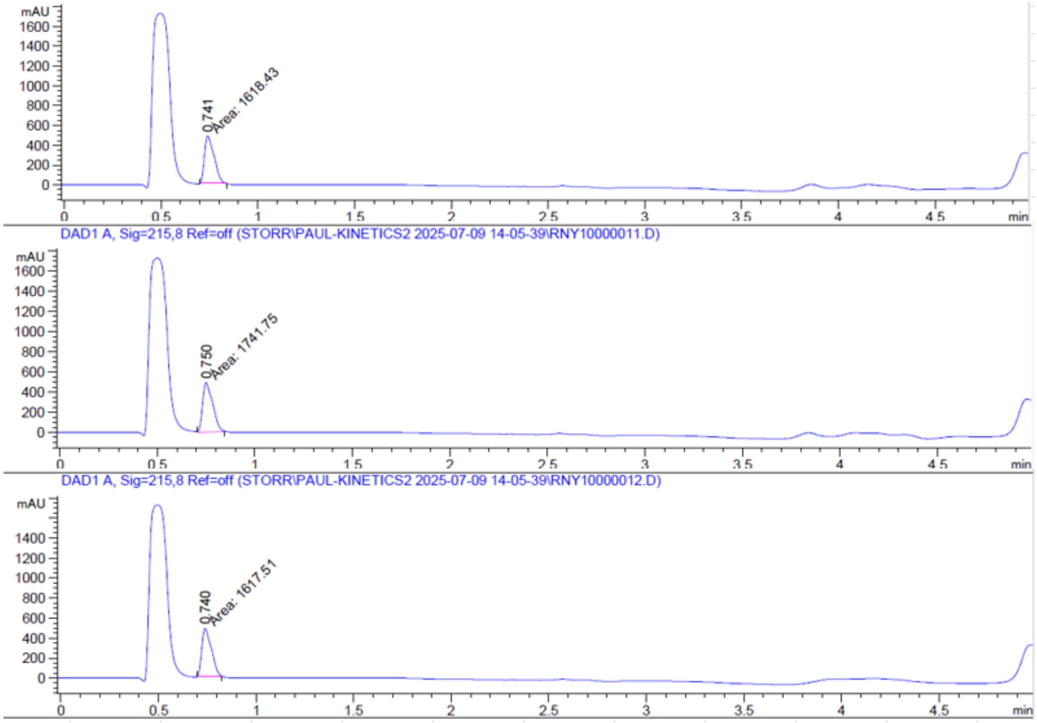

Figure S32. HPLC of **RuP2** photoactivated measured in triplicate, in PBS (0.01 M, pH 7.4). Conditions: [Asc] = 200 μM, [CCA] = 200 μM, [$CuCl_2$] = 25 μM, [RuP2] = 50 μM, [$A\beta_{1\text{-}16}$] = 25 μM.

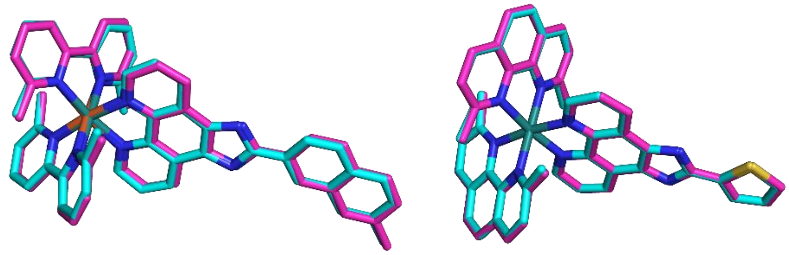

Figure S33. Left: RuP1 (pink carbons B3LYP/LANDL2DZ) and **FeP1** (blue carbons B3LYP/LANDL2DZ) aligned. **Right: RuP2** (pink carbons B3LYP/LANDL2DZ) and **FeP2** (blue carbons B3LYP/LANDL2DZ) aligned. All images show minimal differences in overall geometry.

Table S1. The 9 most stable binding modes with respective binding energy of **RuP1** at 5OQV binding sites (kcal/mol).

**RuP1**	
Mode	Affinity (kcal/mol)
1	-8.891
2	-8.753
3	-8.750
4	-8.617
5	-8.607
6	-8.557
7	-8.507
8	-8.340
9	-8.171

Table S2. The 9 most stable binding modes with respective binding energy of **RuP2** at 5OQV binding sites (kcal/mol).

**RuP2**	
Mode	Affinity (kcal/mol)
1	-7.188
2	-7.155
3	-7.064
4	-7.049
5	-6.904
6	-6.900
7	-6.752
8	-6.731
9	-6.709

Table S3. The 9 most stable binding modes with respective binding energy of **RuP1** at 2MXU binding sites (kcal/mol).

**RuP1**	
Mode	Affinity (kcal/mol)
1	-10.19
2	-10.17
3	-9.377
4	-9.264
5	-8.947
6	-8.79
7	-8.654
8	-8.304
9	-8.312

Table S4. The 9 most stable binding modes with respective binding energy of **RuP2** at 2MXU binding sites (kcal/mol).

**RuP2**	
Mode	Affinity (kcal/mol)
1	-9.119
2	-9.081
3	-8.563
4	-8.539
5	-8.362
6	-8.345
7	-8.319
8	-8.215
9	-7.878

Table S5. HPLC analysis of unactivated and photoactivated **RuP1** and **RuP2** showing the concentration of 3-CCA detected in each trial along with the corresponding average values and standard deviation.

**Compound**	**Trial 1**	**Trial 2**	**Trial 3**	**Average ± SD**
**RuP1** Unactivated	88 μM	84 μM	82 μM	85 ± 3 μM
**RuP1** Activated	215 μM	214 μM	205 μM	212 ± 6 μM
**RuP2** Unactivated	68 μM	73 μM	78 μM	73 ± 5 μM
**RuP2** Activated	212 μM	229 μM	212 μM	218 ± 10 μM

Table S6. HPLC analysis of unactivated and photoactivated **RuP1** and **RuP2** showing the concentration of 7-OH-3-CCA detected in each trial along with the corresponding average values and standard deviation.

Compound	Trial 1	Trial 2	Trial 3	Average ± SD
**RuP1** Unactivated	105 μM	111 μM	111 μM	109 ± 3 μM
**RuP1** Activated	0 μM	0 μM	0 μM	0 μM
**RuP2** Unactivated	120 μM	118 μM	116 μM	118 ± 2 μM
**RuP2** Activated	0 μM	0 μM	0 μM	0 μM

Notes and References

1 J. A. Roque Iii, H. D. Cole, P. C. Barrett, L. M. Lifshits, R. O. Hodges, S. Kim, G. Deep, A. Francés-Monerris, M. E. Alberto, C. G. Cameron and S. A. McFarland, *J. Am. Chem. Soc.*, 2022, **144**, 8317–8336.

2 H. D. Cole, J. A. Roque III, L. M. Lifshits, R. Hodges, P. C. Barrett, D. Havrylyuk, D. Heidary, E. Ramasamy, C. G. Cameron, E. C. Glazer and S. A. McFarland, *Photochem. Photobiol.*, 2022, **98**, 73–84.

3 B. Zhang-Haagen, R. Biehl, L. Nagel-Steger, A. Radulescu, D. Richter and D. Willbold, *PLOS ONE*, 2016, **11**, e0150267.

4 B. S. Howerton, D. K. Heidary and E. C. Glazer, *J. Am. Chem. Soc.*, 2012, **134**, 8324–8327.

5 E. Wachter, D. K. Heidary, B. S. Howerton, S. Parkin and E. C. Glazer, *Chem. Commun.*, 2012, **48**, 9649.

6 A new type of DNA "light-switch": a dual photochemical sensor and metalating agent for duplex and G-quadruplex DNA - Chemical Communications (RSC Publishing), https://pubs.rsc.org/en/content/articlelanding/2014/cc/c3cc47269h, (accessed December 28, 2024).

7 A. N. Hidayatullah, E. Wachter, D. K. Heidary, S. Parkin and E. C. Glazer, *Inorg. Chem.*, 2014, **53**, 10030–10032.

8 *ACS Chem. Neurosci.*, 2024, **15**, 86–97.

9 Ramamoorthy Group - Amyloids, https://rams.biop.lsa.umich.edu/research/amyloids, (accessed December 28, 2024).

10 A. M. Mancino, S. S. Hindo, A. Kochi and M. H. Lim, *Inorg. Chem.*, 2009, **48**, 9596–9598.

11 N. P. Cook, M. Ozbil, C. Katsampes, R. Prabhakar and A. A. Martí, *J. Am. Chem. Soc.*, 2013, **135**, 10810–10816.

12 X. Wang, X. Wang, C. Zhang, Y. Jiao and Z. Guo, *Chem. Sci.*, 2012, **3**, 1304–1312.

13 AutoDock Vina 1.2.0: New Docking Methods, Expanded Force Field, and Python Bindings | Journal of Chemical Information and Modeling, https://pubs.acs.org/doi/10.1021/acs.jcim.1c00203, (accessed May 7, 2025).

14 L. Gremer, D. Schölzel, C. Schenk, E. Reinartz, J. Labahn, R. B. G. Ravelli, M. Tusche, C. Lopez-Iglesias, W. Hoyer, H. Heise, D. Willbold and G. F. Schröder, *Science*, 2017, **358**, 116–119.

15 Y. Xiao, B. Ma, D. McElheny, S. Parthasarathy, F. Long, M. Hoshi, R. Nussinov and Y. Ishii, *Nat. Struct. Mol. Biol.*, 2015, **22**, 499–505.
